# Characterisation of the hepatitis B virus cross-species transmission pattern via Na^+^/taurocholate co-transporting polypeptides from 11 New World and Old World primate species

**DOI:** 10.1371/journal.pone.0199200

**Published:** 2018-06-18

**Authors:** Simon F. Müller, Alexander König, Barbara Döring, Dieter Glebe, Joachim Geyer

**Affiliations:** 1 Institute of Pharmacology and Toxicology, Biomedical Research Center Seltersberg, Justus Liebig University Giessen, Giessen, Germany; 2 Institute of Medical Virology, Biomedical Research Center Seltersberg, Justus Liebig University Giessen, Giessen, Germany; Indiana University, UNITED STATES

## Abstract

The hepatic Na^+^/taurocholate co-transporting polypeptide (NTCP in man, Ntcp in animals) is the high-affinity receptor for the hepatitis B (HBV) and hepatitis D (HDV) viruses. Species barriers for human HBV/HDV within the order Primates were previously attributed to Ntcp sequence variations that disable virus-receptor interaction. However, only a limited number of primate Ntcps have been analysed so far. In the present study, a total of 11 Ntcps from apes, Old and New World monkeys were cloned and expressed *in vitro* to characterise their interaction with HBV and HDV. All Ntcps showed intact bile salt transport. Human NTCP as well as the Ntcps from the great apes chimpanzee and orangutan showed transport-competing binding of HBV derived myr-preS1-peptides. In contrast, all six Ntcps from the group of Old World monkeys were insensitive to HBV myr-preS1-peptide binding and HBV/HDV infection. This is basically predetermined by the amino acid arginine at position 158 of all studied Old World monkey Ntcps. An exchange from arginine to glycine (as present in humans and great apes) at this position (R158G) alone was sufficient to achieve full transport-competing HBV myr-preS1-peptide binding and susceptibility for HBV/HDV infection. New World monkey Ntcps showed higher sequence heterogeneity, but in two cases with 158G showed transport-competing HBV myr-preS1-peptide binding, and in one case (*Saimiri sciureus*) even susceptibility for HBV/HDV infection. In conclusion, amino acid position 158 of NTCP/Ntcp is sufficient to discriminate between the HBV/HDV susceptible group of humans and great apes (158G) and the non-susceptible group of Old World monkeys (158R). In the case of the phylogenetically more distant New World monkey Ntcps amino acid 158 plays a significant, but not exclusive role.

## Introduction

The hepatic Na^+^/taurocholate co-transporting peptide (NTCP in man, Ntcp in animals, gene name: *SLC10A1/Slc10a1*) together with the closely related intestinal apical sodium-dependent bile acid transporter ASBT/Asbt are essential drivers of the enterohepatic circulation of bile salts in mammals [1]. However, only NTCP is exploited by the human hepatitis B (HBV) and hepatitis D (HDV) viruses as high-affinity binding and entry receptor for productive infection of hepatocytes [2–4]. Since NTCP/Ntcp is mainly expressed in hepatocytes [5], the strict liver tropism of HBV and HDV can mainly be explained by organ-specific expression of this receptor protein.

HBV is considered to be a major global health problem with ~250 million chronic HBV carriers and ~1 million HBV-associated human dealths per year [6]. Within the viral family of *Hepadnaviridae* HBV represents the prototype species of the genus *Orthohepadnavirus* infecting mammals. Related hepadnaviruses have been isolated from nonhuman primates, including chimpanzee, gorilla, orangutan, and gibbon [7]. Whereas in the closely related primate family of *Cercopithecidae* (Old World monkeys, OWM, i.e. macaques and baboons) a specific endogenous hepadnavirus has not been identified so far (despite the sporadic finding of HBV in a *Macaca fascicularis* colony from Mauritius, see below), two hepadnavirus species have been detected in more distantly related families of *Platyrrhini*, representing New World monkeys (NWM). Woolly monkey HBV (WMHBV) was originally isolated from a captive woolly monkey (*Lagothrix lagotricha*, family *Atelidae*) with fulminant hepatitis [8] and very recently capuchin monkey HBV (CMHBV) was identified in *Sapajus xanthosternos* (family *Cebidae*) during systematic screening of NWM for novel hepadnaviruses in Brasil [9].

HBV is a small enveloped DNA virus. The partially double-stranded genome of approximately 3.2 kb is coding for large (LHBs), middle (MHBs) and small (SHBs) HBV surface proteins [10]. Among them, the myristoylated preS1-peptide (myr-preS1), comprising the 2–48 N-terminal amino acids of the LHBs, is most relevant for the species-specific infection of hepatocytes [11]. HDV is an enveloped RNA virus and is only found in humans. HDV is a defective virus and requires HBV surface proteins for envelopment, secretion into blood, and infection of hepatocytes via NTCP [12]. Based on the current knowledge, the following factors are relevant for the early entry phase of HBV/HDV infection: (I) low affinity attachment to hepatic heparan sulfate proteoglycans [10], (II) myr-preS1-mediated high-affinity binding to NTCP/Ntcp [2], and (III) viral entry via internalisation of the virus/receptor complex [13]. Whereas the precise sequence of the viral preS1-peptide seems to be only relevant for (II), separate domains of the host NTCP/Ntcp receptor allow or reject virus binding (II) and entry (III) [2,3,14].

Several interspecies transmissions between man and closely-related genera of *Hominoidea* (*Pan*, *Gorilla*, *Pongo*, and *Hylobates*) have been reported, e.g. evidence of HBV from gibbon and human origin in chimpanzees [7]. Although the Ntcp receptor sequence from the apes has not been explicitely investigated so far, this finding is most likely based on high sequence homology among the viral preS1-peptides as well as the NTCP/Ntcp receptor proteins of apes and humans.

In contrast, macaques and baboons were generally regarded as unsusceptible to HBV infection. But, sporadic detection of HBV in these OWMs was reported, e.g. presence of a specific human HBV subgenotype in a *Macaca fascicularis* colony from Mauritius Island [15]. In this study the authors claim that heptocytes from these Mauritius macaques could not be infected with wild type human HBV, but only with the special HBV isolate from the infected Mauritius macaques [15]. In contrast, in another study hepatocytes and Ntcps from *Macaca fascicularis* and *Macaca mulatta* were neither able to bind the HBV myr-preS1-peptide nor mediate HBV infection [2,16]. This might indicate that the mutations found within the preS1 sequence of the Mauritius HBV isolate adapted to the otherwise HBV insensitive macaque Ntcp. However, this hypothesis could not be confirmed since neither the experimentally reconstructed Mauritius macaque HBV strain nor HBV wild type strains could infect hepatocytes from Mauritian cynomolgus macaques *in vivo* or *in vitro* [17].

For the NWM hepadnavirus from woolley monkey (WMHBV) interspecies transmission was clearly demonstrated. Apart from woolley monkey, also spider monkey (*Ateles geoffroyi*) and primary hepatocytes from the more distant *Tupaia belangeri* (asian treeshrew) are susceptible to WMHBV infetion as well as primary chimpanzee and human hepatocytes *in vitro*, but with much lower efficiencies [8,18,19]. The molecular determinants relevant for the interspecies transmission between the *Hominoidea* primates and the NWMs were not completely elucidated so far, in particular as only limited information is available for OWM and NWM Ntcps.

In order to more systematically investigate potential species barriers for viral transmission in primates, 11 Ntcps from representative species of different primate families (apes, OWM, NWM) were cloned and characterised for bile salt transport function. Furthermore, their susceptibility for virus binding and infection was investigated with two representative orthohepadnavirus species circulating in the families of *Hominoidea* (human HBV) and NWM (WMHBV).

## Materials and methods

### Cloning of primate Ntcp cDNAs

RNA extraction from primate liver tissue (commercially obtained from the German Primate Center, Göttingen) was performed with the trizol method after rotor-stator homogenization. Based on a sequence alignment of known and predicted monkey Ntcp cDNAs, primers were selected for full-length PCR amplification of the entire monkey Ntcp ORFs (see Table 1).

**Table 1:** Primers used for *de novo* cloning of monkey Ntcps.

Species abbr.	GenBank Accession No.*	Dir.	Primer sequence (5’ → 3’)**
S.spe	KT326157	for rev	TGT CCT GAG AGG TGA TTA AAG AAG TTT TGC TGC TCT CTS TAG TTK ACC
P.ham	KT382283	for rev	TGT CCT GAG AGG TGA TTA AAG AAG TTT TGC TGC TCT CTS TAG TTK ACC
C.aet	KT382281	for rev	TGT CCT GAG AGG TGA TTA AAG AAG TTT TGC TGC TCT CTS TAG TTK ACC
M.mul	KT382282	for rev	TGT CCT GAG AGG TGA TTA AAG AAG TTT TGC TGC TCT CTS TAG TTK ACC
M.sil	KT326156	for rev	TGT CCT GAG AGG TGA TTA AAG AAG TTT TGC TGC TCT CTS TAG TTK ACC
M.fas	KT382286	for rev	TGT CCT GAG AGG TGA TTA AAG AAG TTT TGC TGC TCT CTS TAG TTK ACC
S.sci	KR153328	for rev	TGT CCT GAG AGG TGA TTA AAG AAG TTT TGC TGC TCT CTS TAG TTK ACC
C.jac	KT382285	for rev	TGT CCT GAG AGG TGA TTA AAG AAG GGC CAA GGC TTG ATG ATT GC
S.oed	KT382284	for rev	TGT CCT GAG AGG TGA TTA AAG AAG CAC AAA ACT TGA AAT GGG ATT TGG G

*All cloned sequences were deposited into the DDBJ/ENA/GenBank database with the indicated Accession numbers.

**In part degenerated primers were used with S representing G or C and K representing G or T.

Touch-down PCR with Fast Start high-fidelity polymerase (Roche) consisted of an initial denaturation of 95°C for 2 min, followed by ten loops of a touch-down circle consisting of denaturation (95°C, 30 s), annealing (58°C—0.5°C per step, 30 s), and elongation (72°C, 80s). After the touch-down phase 20 cycles with an annealing temperature of 53°C were performed, followed by final elongation at 72°C for 6 min. All amplified Ntcp ORFs were cloned into the pcDNA5/FRT/TO vector (Thermo Fisher Scientific) and subjected to DNA sequencing. Site-directed mutagenesis was performed on the cloned human NTCP and monkey Ntcp cDNAs, with the primers listed in Table 2. Correct constructs were selected based on DNA sequencing and were used for transfection into HepG2 and HEK293 cells.

**Table 2:** Primers used for mutagenesis of human NTCP and monkey Ntcps.

Species abbr.	Mutation	GenBank Acc. No.*	Dir.	Primer sequence (5’ → 3’)
H.sap	G158R	NM_003049	for	CAA GGT GCC CTA TAA ACG CAT CGT GAT ATC ACT G
			rev	CAG TGA TAT CAC GAT GCG TTT ATA GGG CAC CTT G
H.sap	G158D	NM_003049	for	GAC AAG GTG CCC TAT AAA GAC ATC GTG ATA TCA CTG
			rev	CAG TGA TAT CAC GAT GTC TTT ATA GGG CAC CTT GTC
H.sap	G158N	NM_003049	for	GAC AAG GTG CCC TAT AAA AAC ATC GTG ATA TCA CTG G
			rev	CCA GTG ATA TCA CGA TGT TTT TAT AGG GCA CCT TGT C
H.sap	G158V	NM_003049	for	CAA GGT GCC CTA TAA AGT CAT CGT GAT ATC ACT GG
			rev	CCA GTG ATA TCA CGA TGA CTT TAT AGG GCA CCT TG
H.sap	G158S	NM_003049	for	GAA GGA CAA GGT GCC CTA TAA AAG CAT CGT GAT ATC
			rev	GAT ATC ACG ATG CTT TTA TAG GGC ACC TTG TCC TTC
P.abe	A6V	NM_003049	for	GAG GCC CAC AAC GTG TCT GCC CCA TTC
			rev	GAA TGG GGC AGA CAC GTT GTG GGC CTC
P.abe	I303M	NM_003049	for	GGC TTC TCC TCA TTG CCA TGT TTT GGT GCT ATG AG
			rev	CTC ATA GCA CCA AAA CAT GGC AAT GAG GAG AAG CC
P.abe	I320T	NM_003049	for	CCA AGG ATA AAA CAA AAA TGA CCT ACA CAG CTG CCA C
			rev	GTG GCA GCT GTG TAG GTC ATT TTT GTT TTA TCC TTG G
P.tro	K157R	NM_003049	for	GAA GGA CAA GGT GCC CTA TAG AGG CAT CGT G
			rev	CAC GAT GCC TCT ATA GGG CAC CTT GTC CTT C
P.tro	I303M	NM_003049	for	GGC TTC TCC TCA TTG CCA TGT TTT GGT GCT ATG AG
			rev	CTC ATA GCA CCA AAA CAT GGC AAT GAG GAG AAG CC
P.tro	I315G	NM_003049	for	GAG AAA TTC AAG ACT CCC AAG GGT AAA ACA AAA ATG ATC TAC AC
			rev	GTG TAG ATC ATT TTT GTT TTA CCC TTG GGA GTC TTG AAT TTC TC
S.spe	R158G	KT326157	for	CAA GGT GCC CTA TGG AGG CAT CAT ATT ATC ACT G
			rev	CAG TGA TAA TAT GAT GCC TCC ATA GGG CAC CTT G
P.ham	R158G	KT382283	for	CAA GGT GCC CTA TGG AGG CAT CAT ATT ATC ACT G
			rev	CAG TGA TAA TAT GAT GCC TCC ATA GGG CAC CTT G
C.aet	R158G	KT382281	for	CAA GGT GCC CTA TGG AGG CAT CAT ATT ATC ACT G
			rev	CAG TGA TAA TAT GAT GCC TCC ATA GGG CAC CTT G
M.mul	R158G	KT382282	for	CAA GGT GCC CTA TGG AGG CAT CAT ATT ATC ACT G
			rev	CAG TGA TAA TAT GAT GCC TCC ATA GGG CAC CTT G
M.sil	R158G	KT326156	for	CAA GGT GCC CTA TGG AGG CAT CAT ATT ATC ACT G
			rev	CAG TGA TAA TAT GAT GCC TCC ATA GGG CAC CTT G
M.fas	R158G	KT382286	for	CAA GGT GCC CTA TGG AGG CAT CAT ATT ATC ACT G
			rev	CAG TGA TAA TAT GAT GCC TCC ATA GGG CAC CTT G
S.sci	G158R	KR153328	for	CAA GGT GCC CTA TGG ACG CAT CAT GAT ATC ACT G
			rev	CAG TGA TAT CAT GAT GCG TCC ATA GGG CAC CTT G
S.sci	G158S	KR153328	for	CAA GGT GCC CTA TGG AAG CAT CAT GAT ATC ACT G
			rev	CAG TGA TAT CAT GAT GCT TCC ATA GGG CAC CTT G
C.jac	G158R	KT382285	for	GAC AAG GTG CCC TAT AAA CGC ATC ATG AGA TCG C
			rev	GCG ATC TCA TGA TGC GTT TAT AGG GCA CCT TGT C
C.jac	G158S	KT382285	for	GAC AAG GTG CCC TAT AAA AGC ATC ATG AGA TCG C
			rev	GCG ATC TCA TGA TGC TTT TAT AGG GCA CCT TGT C
S.oed	S158G	KT382284	for	GAA GGA CAA GGT GCC CTA TAA AGG CAT CAT GAT ATC
			rev	GAT ATC ATG ATG CCT TTA TAG GGC ACC TTG TCC TTC

*Reference sequence that was used for mutagenesis.

### Cell culture of HEK293 and HepG2 cells and transfection

GripTite 293 MSR cells (further referred to as HEK293 cells, Invitrogen), a modified HEK293 cell line expressing human macrophage scavenger receptor for stronger adhesion, were maintained at 37°C, 5% CO_2_ and 95% humidity in DMEM/F-12 medium (Thermo Fisher Scientific) supplemented with 10% fetal calf serum (Sigma), 4 mM L-glutamine (PAA) and penicillin/streptomycine (PAA). HepG2 cells (Clontech) were cultivated under the same conditions in DMEM medium with all supplements listed above, except for L-glutamine. HEK293 cells were transiently transfected with human NTCP and monkey Ntcp constructs for all transport and peptide binding assays. Transfection was performed with Lipofectamine 2000 (Thermo Fisher Scientific). NTCP/Ntcp-transfected HepG2 cells were used for all HBV/HDV infection experiments as reported before [4]. They were transfected using X-tremeGENE (Sigma-Aldrich).

### Bile salt transport assays

Qualitativ transport experiments were performed in NTCP/Ntcp-transfected HepG2 cells with the fluorescent bile salt 4-nitrobenzo-2-oxa-1,3-diazole taurocholate [20] (NBD-TC) in DMEM. Nuclei were stained with Hoechst33342 and transport was assessed on Leica DMI6000 B inverted fluorescent microscope. Quantitative transport measurements were performed with [^3^H]taurocholate ([^3^H]TC, Perkin Elmer) in HEK293 cells as reported before [21]. N-terminally myristoylated peptides consisting of amino acids 2–48 of the preS1 domain of HBV or WMHBV with additional C-terminal strep-tag (further referred to as myr-preS1-peptide) were purchased from Biosynthesis (Lewisville, Texas, USA) and used for [^3^H]TC transport inhibition as reported before [4].

### HBV and WMHBV myr-preS1-Al633-peptide binding

HBV and WMHBV myr-preS1-peptides C-terminally labelled with AlexaFluor633 (further referred to as myr-preS1-Al633, Biosynthesis) were used for direct binding assays in NTCP/Ntcp-transfected HEK293 cells as described before [4]. The sodium-dependent organic anion transporter (SOAT), unable to transport TC [21] and unable to bind the myr-preS1-peptide [4], served as negative control. HEK293 cells were plated in 24-well plates and incubated for 20 min with 250 ml of DMEM containing 10 nM of the HBV or WMHBV myr-preS1-Al633-peptides at 37°C. After a washing step with 300 µl DMEM at 37°C for 2 min, cells were covered with 300 µl DMEM and plates were scanned at room temperature with a Typhoon FLA 7000 fluorescence reader (GE Healthcare Life Sciences) with the following settings: fluorescence mode with normal detection, 633 nm laser for excitation, focus level +3 mm, 100 µm resolution, 700 V currency of the photomultiplier tube and 670 ± 30 nm band-pass emission filter. The total signal per area was determined by Image Quant Software.

### Infection of HepG2 cells with HBV/HDV

NTCP/Ntcp-transfected HepG2 cells were inoculated for 16 h with similar genome equivalents/cell of HDV particles pseudotyped with HBV or WMHBV envelopes (HDVpsHBV/HDVpsWMHBV). HBV was produced *in vitro* as described [4]. Infection was done in hepatocyte growth medium (HGM) supplemented with 2% DMSO and 4% polyethylenglycol as reported before [4]. Thereafter, cells were washed twice with HGM and cultured until day 10 post infection in HGM supplemented with 2% DMSO and 2% FCS. Fixation was performed at 10 days post infection with 3.7% formaldehyde and 1% methanol at 4°C for 30 min. Cells were permeabilized with 0.2% Triton X100 in phosphate buffered saline (PBS, containing 137 mM NaCl, 2.7 mM KCl, 1.5 mM KH_2_PO_4_, 7.3 mM Na_2_HPO_4_, pH 7.4) for 20 min at room temperature. Unspecific binding epitopes were blocked by incubation with 10% fetal calf serum in PBS for 45 min at 37°C. For detection of HBV core (HBc) protein expression, cells were incubated for 2 h at 37°C with a polyclonal rabbit anti-HBcAg antiserum (1:500 dilution, Dako, Hamburg, Germany) in PBS and thereafter with anti-rabbit IgG AlexaFluor594 (1:200 dilution in PBS, Immuno Jackson) for 1 h at 37°C. For detection of the HDV antigen (HDAg) as a marker for HDV infection, cells were incubated with a 1:100 dilution of purified IgG preparation of an HDV/HBV coinfected person, followed by incubation with anti-human IgG AlexaFluor594 (Immuno Jackson) diluted 1:200 in PBS for 1 h at 37°C. Nuclei were stained with DAPI (10 µg/ml) in PBS.

### Homology modelling

For 3D homology modelling the human NTCP and *Macaca mulatta* Ntcp protein sequences were used as queries for the SWISS-MODEL tool (https://swissmodel.expasy.org). Models were calculated based on the template of ASBT from *Yersinia frederiksenii* (ASBT_Yf_, PDB 4n7w, sequence identity to NTCP/Ntcp of 26%) [22]. The original structure of the bacterial protein demonstrates 10 transmembrane segments, however with an extended N-terminus forming transmembrane segment 1, which is not present in the human NTCP protein. Therefore, based on sequence alignment the sequence homologous transmembrane segments were allocated (ASBT_Yf_ → NTCP) as follows: 2→1, 3→2, 4→3, 5→4, 6→5, 7→6, 8→7, 9→8, and 10→9. Overall, localisation of the transmembrane segments also fits very well with the *de novo* predictions of several algorithms, including HMMTOP (http://www.enzim.hu/hmmtop/) and MEMSAT3 (http://bioinf.cs.ucl.ac.uk/software_downloads/memsat/). The N-terminus of NTCP/Ntcp was oriented to the outside and the C-terminus to the intracellular site. The N-termini (amino acids 1 to 26 for NTCP and amino acids 1 to 27 for M.mul Ntcp) and the C-termini (amino acids 309 to 349 for both) could not be structurally predicted and so are not shown in the 3D models. Therefore, the models represent amino acids 27 to 308 (out of 349) for human NTCP and amino acids 28 to 308 (out of 349) for M.mul Ntcp.

## Results

### Cloning of NWM and OWM Ntcps

**Fig 1:**
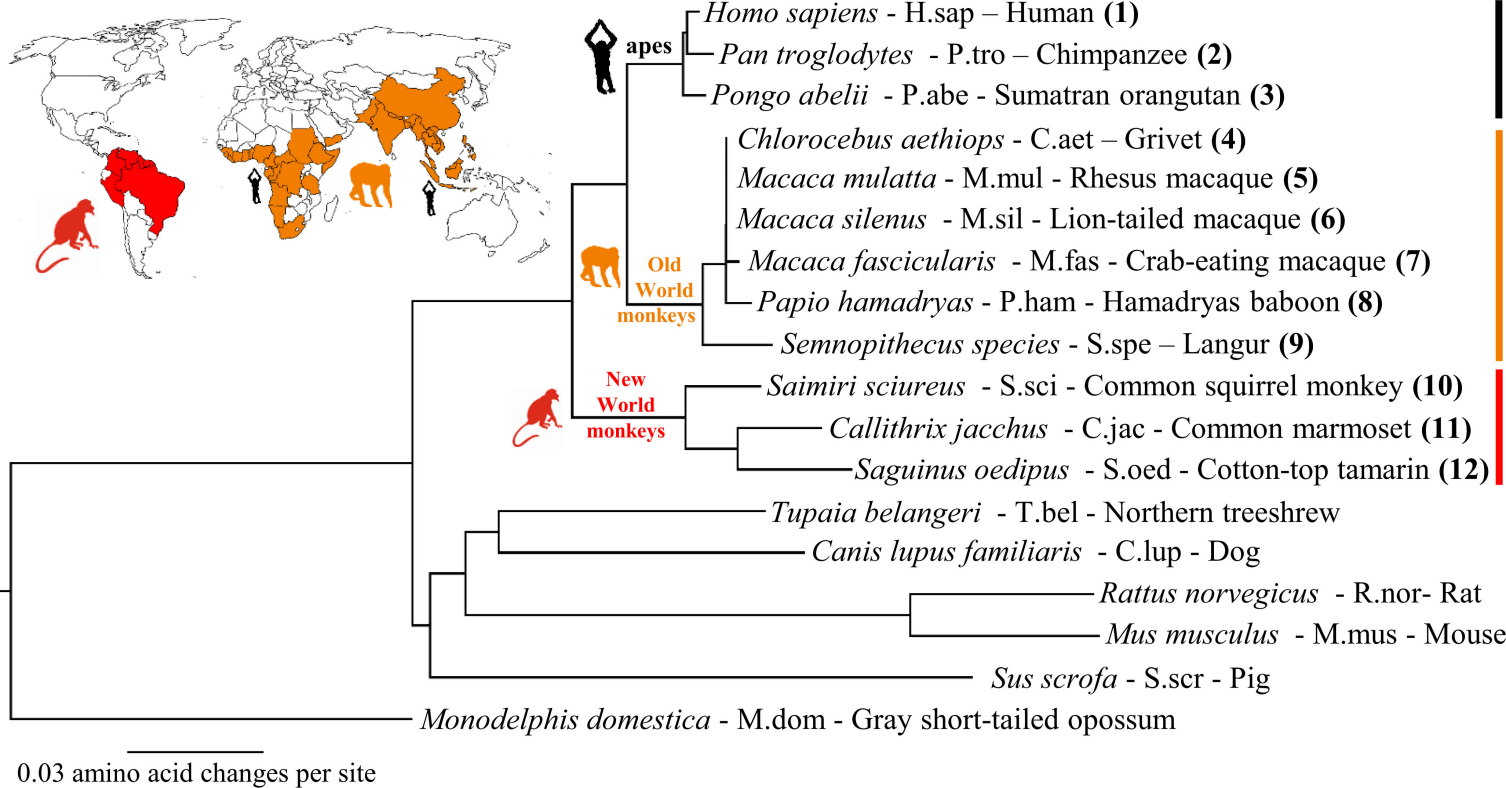
Phylogenetic classification of the cloned monkey Ntcps. Phylogenetic relationship of monkey Ntcps with their habitats in the Old World (orange) or New World (red). Abbreviations, given after the species names, are used instead of the full species names throughout the manuscript. NTCP/Ntcps from non-primate species are included for comparison. Tree-outgroup: *Monodelphis domestica* (M.dom).

A GenBank/ENA/DDBJ database search performed at the beginning of this project revealed cloned monkey Ntcp transcripts only for *Macaca fascicularis* (GenBank Accession No. AK240619.1). A small number of other primate Ntcps were only available as predicted sequences, e.g. from *Papio anubis* (GenBank Accession No. XM_003901980.2). At this date there was no experimentally cloned Ntcp sequence available from any NWM. Thus, Ntcp transcripts were *de novo* cloned from a series of liver samples, derived from six species with habitats in the Old World (*Macaca mulatta*, M.mul; *Macaca fascicularis*, M.fas; *Macaca silenus*, M.sil; *Semnopithecus sp*., S.spe; *Papio hamadryas*, P.ham; and *Chlorocebus aethiops*, C.aet) and three monkeys with habitats in the New World (*Saimiri sciureus*, S.sci; *Callithrix jacchus*, C.jac; and *Saguinus Oedipus*, S.oed) (Fig 1). Liver samples of apes were not available and those Ntcps were generated by mutagenesis based on the available genetic sequence information for chimpanzee (*Pan troglodytes*, P.tro, GenBank Accession No. XM_510035) and orangutan (*Pongo abelii*, P.abe, GenBank Accession No. XM_002824890) with human NTCP as the template sequence. All *de novo* cloned monkey Ntcp sequences were deposited into the GenBank/ENA/DDBJ database (Table 3). A full sequence alignment of all cloned monkey Ntcps with human NTCP is provided in S1 Fig. OWM Ntcps showed a length of 349 amino acids with high sequence identities of >95% compared to human NTCP (Table 3). NWM Ntcps also showed high sequence identity to human NTCP of 90–91%, but in two cases (C.jac and S.oed) revealed elongated C-terminal ends due to frameshift shift mutation at amino acid position 345. These elongated C-termini do not have any sequence similarity to one of the other (even non-primate) Ntcps at all (see S1 Fig). As expected, the phylogenetic analysis revealed clusters for the NWM Ntcps (red in Fig 1) and the OWM Ntcps (orange in Fig 1). The ape Ntcps clustered with human NTCP. Higher sequence distance was seen for the Ntcps from other non-primate species such as *Tupaia belangeri*, pig, dog, mouse, and rat.

**Table 3:** *De novo* cloned monkey Ntcps.

Species	Species abbr.	Name	Protein length	% aa identity to human NTCP	GenBank Accession No.
*Semnopithecus sp*. (OWM)	S.spe	Langur	349 aa	95.4	KT326157
*Papio hamadryas* (OWM)	P.ham	Hamadryas baboon	349 aa	95.7	KT382283
*Chlorocebus aethiops* (OWM)	C.aet	Grivet	349 aa	96.3	KT382281
*Macaca mulatta* (OWM)	M.mul	Rhesus macaque	349 aa	96.3	KT382282
*Macaca silenus* (OWM)	M.sil	Lion-tailed macaque	349 aa	96.3	KT326156
*Macaca fascicularis* (OWM)	M.fas	Crab-eating macaque	349 aa	96.0	KT382286
*Saimiri sciureus* (NWM)	S.sci	Common squirrel monkey	349 aa	92.0	KR153328
*Callithrix jacchus* (NWM)	C.jac	Common marmoset	387 aa*	90.3** / 91.3***	KT382285
*Saguinus oedipus* (NWM)	S.oed	Cotton-top tamarin	371 aa*	89.4** / 90.4***	KT382284

*Frameshift variant at amino acid 345 of the C-terminal end (see S1 Fig)

**Including the c-terminal frameshift sequence

***Excluding the c-terminal frameshift sequence

**Fig 2:**
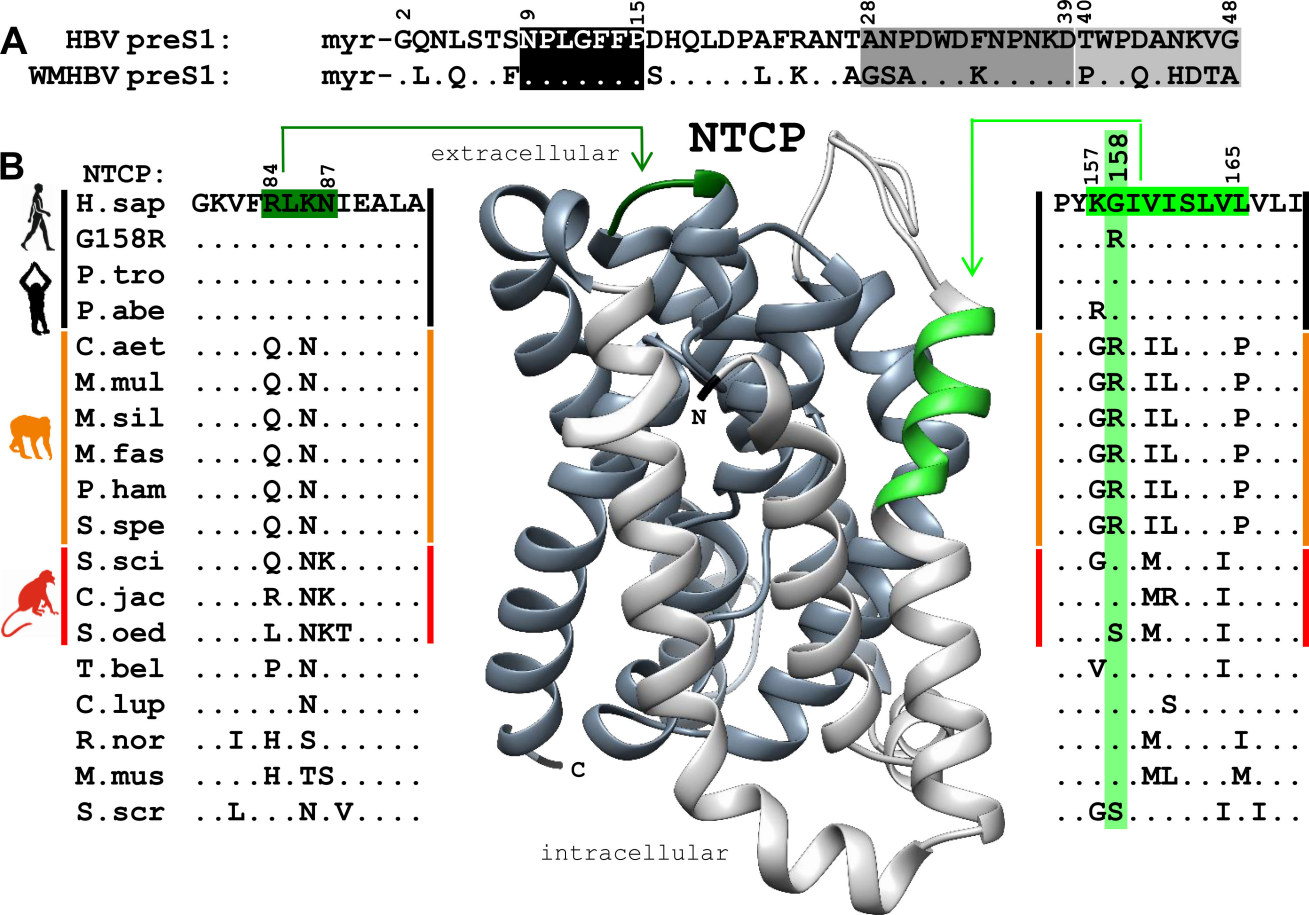
Ntcp regions critical for HBV/WMHBV binding. **(A)** PreS1_2-48_ peptide sequences of human HBV, genotype D, and of woolly monkey HBV (WMHBV). The following domains are supposed to interact with NTCP/Ntcp: main binding domain (black, amino acids 9–15), accessory binding domains I (dark grey, amino acids 28–39) and II (light grey, amino acids 40–48). **(B)** Alignment of Ntcps from apes (black), Old World monkeys (orange), and New World monkeys (red). The alignment is restricted to the regions known to be critical for HBV binding (light green, 84–87) and infection (dark green, 157–165). Both regions are highlighted at a homology model of NTCP.

The myr-preS1-peptide sequences of HBV and WMHBV are shown in Fig 2A. The peptides are supposed to interact with the extracellular part of NTCP/Ntcp via their main binding domains (black box), which are highly conserved between both peptides. In contrast, the domains within the NTCP/Ntcp receptor protein, that were previously identified to be critical for HBV binding and infection (amino acids 84–87 [3,14] and 157–165 [2]), revealed few but significant Ntcp sequence differences between the groups of monkeys. Amino acid position 158 was one of the most prominent distractors between the clusters of apes, OWMs and NWMs. Whereas, glycine (158G) is present in human NTCP and the known ape Ntcps, arginine (158R) is found in all OWM Ntcps. The NWM Ntcps were heterogeneous at this position, with 158G being present in S.sci and C.jac, and with serine (158S) in S.oed (Fig 2B). To analyse the impact of this single amino acid for HBV binding and infection, we generated a series of mutants (G158R, R158G, and some others) on the various NTCP/Ntcps.

### Bile salt transport-competing myr-preS1-peptide binding

**Fig 3:**
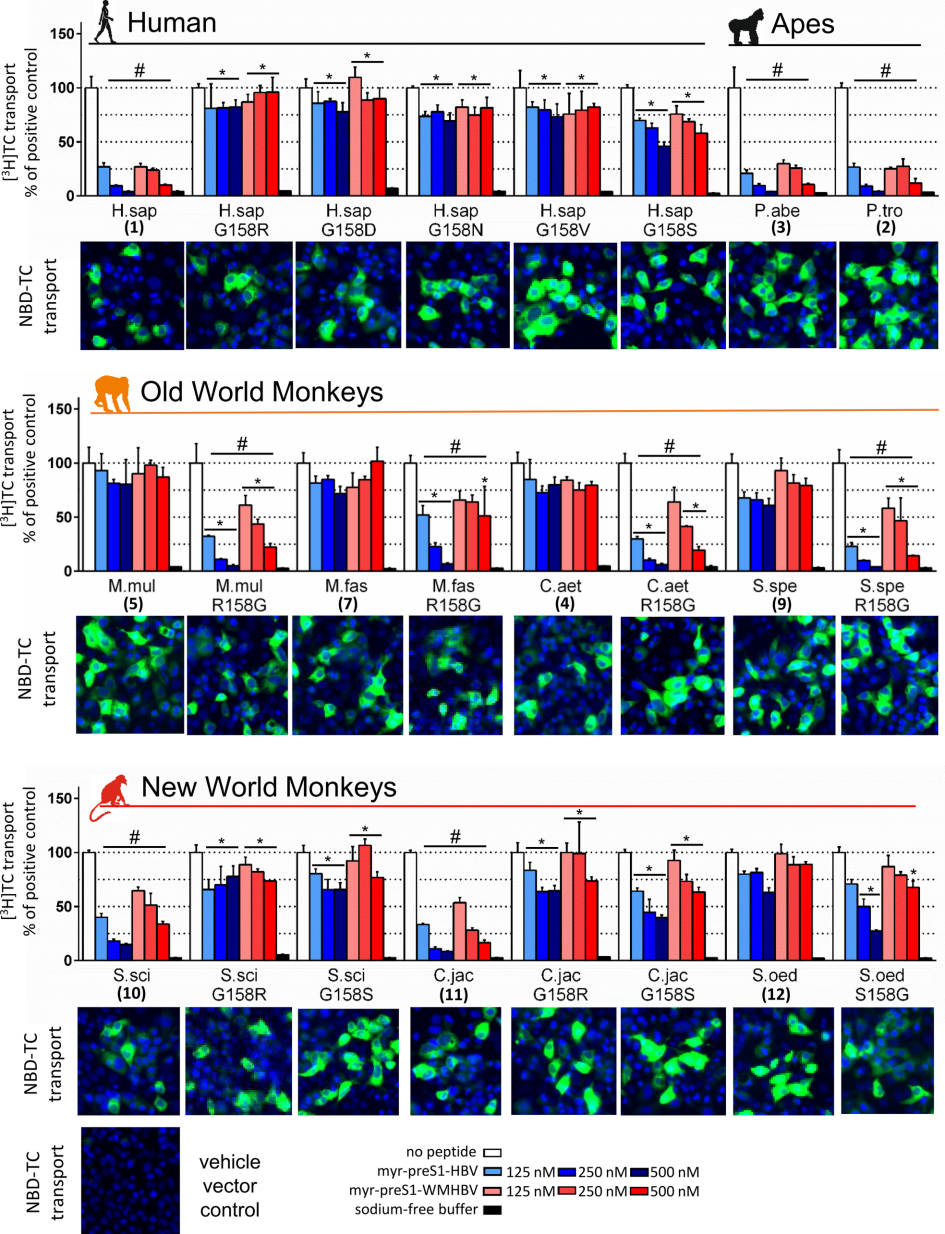
Transport-competing HBV myr-preS1-peptide binding to monkey Ntcps. HEK293 cells were transiently transfected with human/monkey NTCP/Ntcp wild type or mutant constructs. Transport activity was qualitatively verified with NBD-TC (green fluorescence, nuclei blue fluorescence) and was quantitatively measured with [^3^H]TC. Absence of myr-preS1 served as positive control (scaled to 100%, open bars). HBV (blue bars) or WMHBV (red bars) myr-preS1-peptides served as inhibitors at increasing concentrations. Negative control: uptake in sodium-free buffer (black bars). Data represent means ± SD of n = 3 determinations. ^#^Significant transport inhibition compared to positive control, *p*<0.0001. *Significantly different from the corresponding value of the wild type NTCP/Ntcp, *p*<0.001 (two-way ANOVA).

All expressed wild type and mutant NTCP/Ntcp clones qualitatively demonstrated uptake and accumulation of the green fluorescent bile salt NBD-TC (Fig 3), indicating intact transporter function. For quantification, transport assays were additionally performed with [^3^H]TC. All wild type and mutant NTCPs/Ntcps showed comparable transport rates and strictly sodium-dependent transport activity (open bars vs. black bars), and thereby also proved their functional localization at the plasma membrane (Figs 3 and S2).

Specific binding of the HBV myr-preS1-peptide (amino acids 2–48, see Fig 2) to human NTCP is essential for HBV entry and infection, but effective myr-preS1-peptide binding also blocks the physiological bile salt transport activity of NTCP in a concentration-dependent manner [3,4]. Thus, the specificity of viral-host interaction at the level of the receptor NTCP/Ntcp can be indirectly demonstrated by bile salt transport-competing myr-preS1-peptide binding. Therefore, we systematically screened all wild type and mutant NTCP/Ntcps for their TC transport activity at increasing concentrations of the HBV (blue bars) and WMHBV (red bars) myr-preS1-peptides. Interestingly, besides human NTCP, only the two ape Ntcps (P.tro, No.2, and P.abe, No.3) as well as the NWM Ntcps from S.sci (No.10) and C.jac (No.11) showed a pronounced dose-dependent transport inhibition (Fig 3). Of note, all of them have a glycine at position 158. In contrast, TC transport of all OWM Ntcps (Nos.4-9) was not affected by the HBV and WMHBV myr-preS1-peptides at all (Figs 3 and S2). Surprisingly, by swapping of the amino acids 158G and 158R (G158R and *vice versa* R158G), all relevant monkey Ntcps completely inverted their myr-preS1-peptide susceptibility pattern. All OWM R158G Ntcps mutants became susceptible to TC transport inhibition by the myr-preS1-peptides. In contrast, the G158R Ntcp mutants from the NWM S.sci (No.10) and C.jac (No.11) as well as the G158R mutant of human NTCP (No.1) became insensitive against the hepadnaviral myr-preS1-peptides. In order to evaluate if this phenotype conversion is specific for the amino acid exchange from glycine to arginine, a series of further G158X substitutions was analysed, including G158D, G158N, G158V, and G158S. Comparable to G158R mutation, human NTCP lost its susceptibility to myr-preS1-peptide transport inhibition by each of these mutations, indicating that this effect is not specific for arginine (Fig 3, upper panel). Closer analysis of the role of amino acid 158 was also performed for the Ntcp from S.oed (No.12). In this case a serine (158S) is present and the wild type S.oed Ntcp showed to be insensitive against myr-preS1-peptide transport inhibition. However, this sensitivity could be significantly increased by S158G mutation, but not to the level of the other 158G bearing monkey Ntcps.

### Direct binding of HBV and WMHBV myr-preS1-Al633-peptides

**Fig 4:**
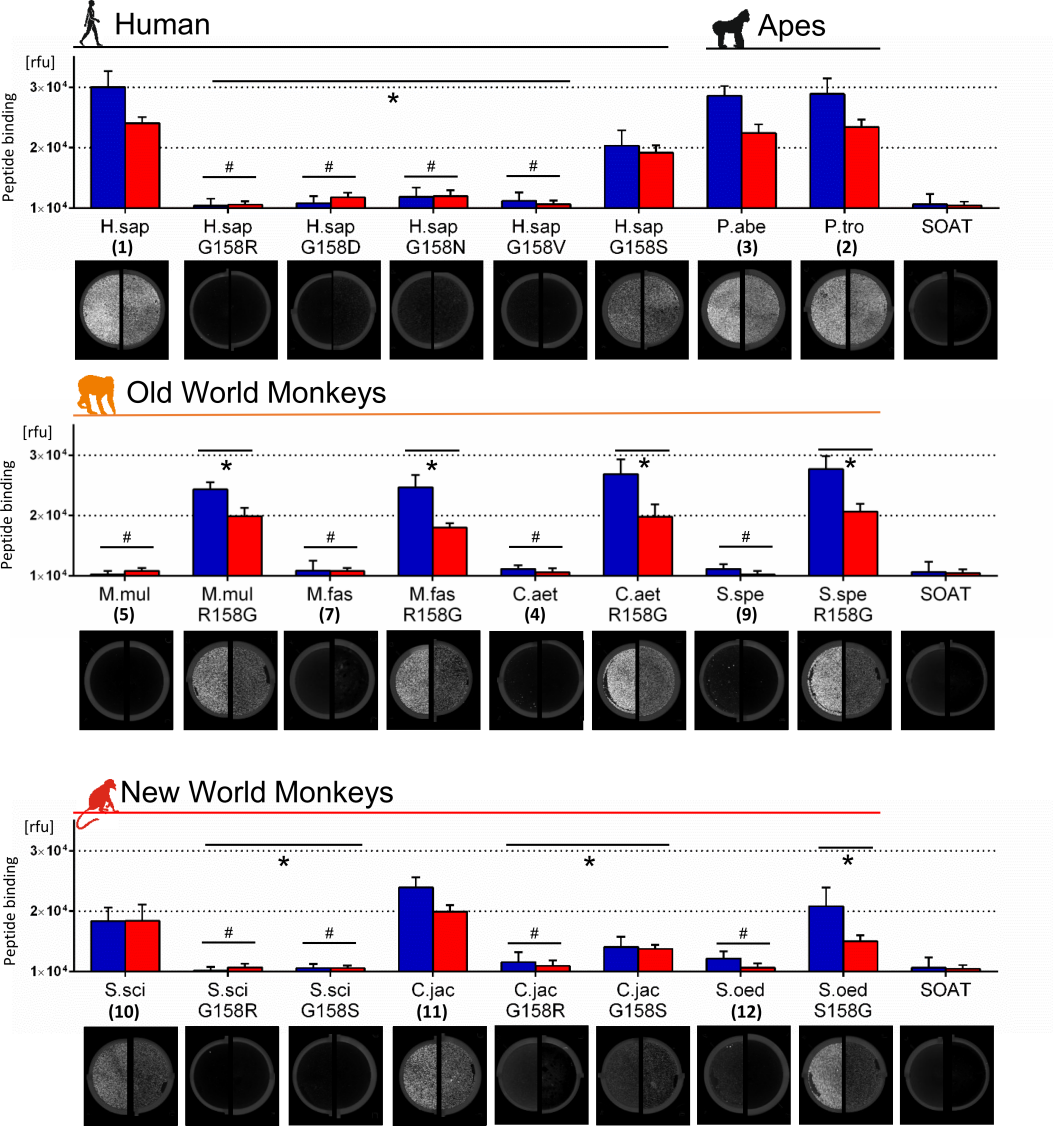
Direct HBV myr-preS1-peptide binding to monkey Ntcps. Human or monkey NTCP/Ntcp-transfected HEK293 cells were incubated with the fluorescently labelled myr-preS1-HBV-Al633 (blue columns) or myr-preS1-WMHBV-Al633 (red columns) peptides (10 nM, 20 min, 37°C). After washing, cells were analysed for Al633 fluorescence. Data represent means ± SD of three combined independent experiments each with triplicate determinations. Representative fluorescence scans are shown for myr-preS1-HBV-Al633 (left half) and myr-preS1-WMHBV-Al633 (right half). SOAT-expressing HEK293 cells served as negative control. ^*^Statistically different from the wild type clone, *p*<0.001. ^#^No significant difference to negative control, *p*<0.001 (two-way ANOVA). Rfu, relative fluorescence units.

In order to directly analyse the specific interaction of HBV and WMHBV with the different NTCP/Ntcps, we quantified binding of HBV and WMHBV myr-preS1-peptides to surface-expressed NTCP/Ntcps using peptides covalently labelled with Alexa633 (Al633) (see Fig 4). Both myr-preS1-Al633 peptides significantly bound to wild type NTCP/Ntcps from human and apes, respectively. The human NTCP mutants G158R, G158D, G158N, and G158V completely lost their HBV (blue columns) and WMHBV (red columns) myr-preS1-peptide binding capacity and were comparable to the unspecific binding control SOAT, which is insensitive to specific myr-preS1-peptide binding and HBV infection [4]. However, G158S mutation of human NTCP revealed an intermediate state, with significant myr-preS1-peptide binding, but at a lower level compared to wild type NTCP. All wild type Ntcps of the OWM (Nos.4-9) did not support any binding of the HBV and WMHBV myr-preS1-peptides at all. In contrast, all R158G mutants gained full HBV and WMHBV myr-preS1-peptide binding capacity to the level of human NTCP and ape Ntcps (Figs 4 and S2). Interestingly, comparable with human NTCP and ape Ntcps, these OWM R158G mutants generally showed higher binding capacity for the HBV-derived myr-preS1-peptide compared to the WMHBV-derived peptide.

In the group of NWMs, data from the transport-competing myr-preS1-peptide binding experiments could be fully confirmed by the direct peptide binding assay. Only the Ntcps from S.sci (No.10) and C.jac (No.11) showed binding capacity for the HBV and WMHBV myr-preS1-Al633 peptides. Of note, Ntcp from S.sci was the only one who showed comparable binding for the myr-preS1-peptides from HBV and WMHBV. In contrast, Ntcp from S.oed (No.12) revealed myr-preS1-Al633-peptide binding at the level of the negative control. Both G158R mutants of the Ntcps from S.sci and C.jac reacted as expected and completely lost their myr-preS1-peptide binding ability. However, there was discrepancy for the G158S mutants: G158S from S.sci did not react, while G158S from C.jac showed significant myr-preS1-peptide binding, but at a lower level compared to wild type C.jac Ntcp (with 158G). In the case of Ntcp from S.oed, S158G mutation significantly increased the myr-preS1-peptide binding levels compared to the wild type (with 158S).

### Infection with HBV or pseudotyped HDV particles

**Fig 5:**
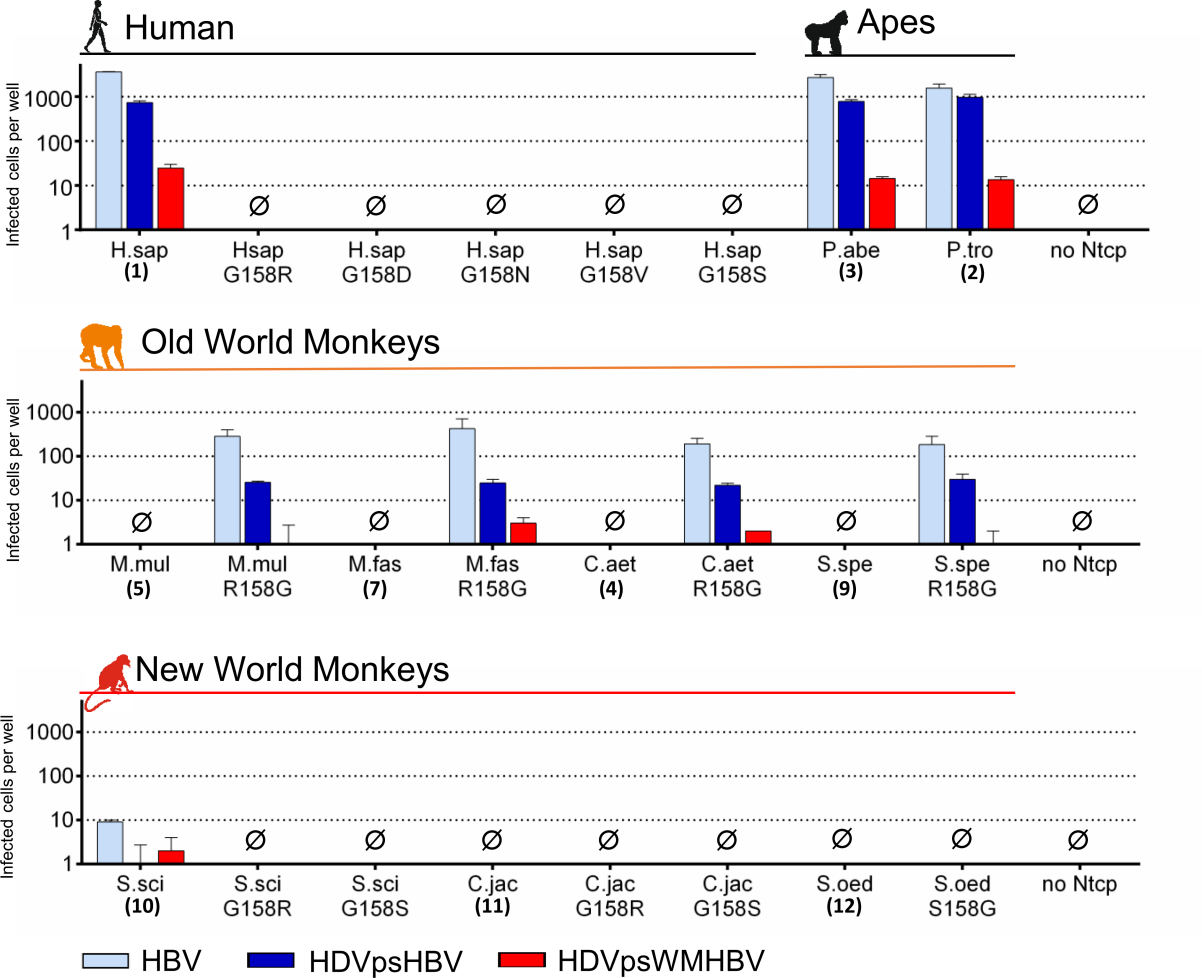
HBV/HDV infection via human and monkey NTCP/Ntcps. HepG2 cells were transiently transfected with human/monkey NTCP/Ntcps wild type or mutant constructs in 96 well plates and inoculated with 10,000 genome equivalents of pseudotyped HDV particles (HDVpsHBV/HDVpsWMHBV) or 2,000 HBV genome equivalents per cell. Cells were cultured for 10 days post infection and then immunostained against HDAg or HBcAg. Infected cells per well were manually counted by fluorescence microscopy. Cell counts are depicted as means ± SD of one representative experiment performed in triplicate.

Finally, the ability of the wild type and mutant Ntcps to support infection with HBV or HDV particles pseudotyped with HBV or WMHBV envelopes (HDVpsHBV/HDVpsWMHBV) was analysed in transiently Ntcp-transfected HepG2 cells (Figs 5 and S2). HBV infection was detected by immunofluorescence against newly produced HBcAg, which increased from day 7 to day 10 post infection (S3 Fig). In contrast, at day 4 post infection, no specific anti-HBcAg immunofluorescence could be detected, demonstrating productive HBV infection of the NTCP-transfected cells. HBV could efficiently infect all cells expressing wild type NTCP and ape Ntcps, while HDV infection generally resulted in lower yield. Of note, HDVpsWMHBV showed up to 100-fold reduced infection rates compared to HDVpsHBV. No infection was observed for any of the NTCP mutants. As expected, HepG2 cells expressing any of the OWM Ntcps (Nos.4-9) were not susceptible for HBV/HDV infection at all. But remarkably, susceptibility to infection was gained with all OWM Ntcps after R158G mutation (Figs 5 and S2). Of note, the infection patterns of HBV, HDVpsHBV and HDVpsWMHBV occurred similar to human NTCP and ape Ntcps, although at an about 10-fold lower rate. Thus, infection with HDVpsWMHBV dropped to the border of detectability and was in some cases either not detectable or below the cut-off level. Interestingly, as the only representative from the group of NWM, Ntcp of S.sci (No.10) revealed a very low, but reproducible susceptibility for HBV infection (Fig 5). In contrast, the two other NWM Ntcps and all of their mutants were unable to support HBV and HDV infection, even not with HDV particles pseudotyped with the envelopes from the NWM WMHBV.

## Discussion

While primate hepadnaviruses are known for the *Hominoidea* superfamily (including human, ape, and gibbon HBV), and the *Platyrrhini* (WMHBV and CMHBV), no endogenous hepadnavirus was isolated from the *Cercopithecoidea* superfamily of OWM so far [7] (despite the above mentioned sporadic detection of HBV in Mauritian cynomolgus macaques [15]). Interspecies transmissions have been described within the *Hominoidea* and *Platyrrhini in vivo*, but are also possible between these groups and beyond *in vitro*, including e.g. infection of human hepatocytes with WMHBV [2,4]. On this background it is surprising that OWM, that are phylogenetically closer to the hominoids than the NWM, seem to be insensitive against HBV and WMHBV binding and infection, at least under experimental conditions [2,16]. This insensitivity was previously attributed to a specific domain of *Macaca fascicularis* Ntcp, representing amino acids 157–165 [2]. But it is not known so far if this domain is present in all OWM Ntcps and if it is also relevant for virus-receptor interaction of the largely unknown NWM Ntcps.

The most striking observation of the present study was that a single amino acid at position 158 of NTCP/Ntcp is sufficient to discriminate between the HBV/HDV susceptible group of humans and apes (all having 158G) and the non-susceptible group of OWM (all having 158R). This amino acid is located within the above mentioned domain [2]. In our experiments, the exchange from 158R to 158G alone was sufficient to achieve full transport-competing myr-preS1-peptide binding and susceptibility for HBV/HDV infection in all analysed R158G Ntcp mutants from OWM. *Vice versa* G158R mutation made human and ape NTCP/Ntcps completely insensitive for myr-preS1-peptide binding and HBV/HDV infection. Of note, NTCP/Ntcp transport activity remained completely unaffected by G158R or R158G mutation. This is a relevant fact, as mutations at receptor or transporter proteins may affect proper membrane insertion or folding, what than would hamper subsequent functional assays. Our experimental setup included three subsequent steps. First, preS1-pepides from HBV and WMHBV were used at increasing concentrations to inhibit the bile salt transport by NTCP/Ntcp. This assay only indirectly measures myr-preS1-peptide binding to NTCP/Ntcp, but allows to verify functional transporter expression in the plasma membrane within the same assay [4]. Second, direct myr-preS1-peptide binding experiments were performed with fluorescently labelled peptides of HBV and WMHBV and, finally infection experiments were added with HBV as well as HDV particles pseudotyped with the surface proteins of HBV and WMHBV. Based on these experiments on six different OWM Ntcps, arginine at position 158 can be regarded as the main source for hepadnavirus insensitivity of the *Cercopithecidae* (S4 Fig). This conclusion is supported by findings from another recent study, showing that Ntcps as well as primary hepatocytes from M.fas and M.mul are insensitive against myr-preS1-peptide binding and HBV/HDV infection [16]. However, transduction of human NTCP into primary hepatocytes from these macaques made them fully susceptible for HBV and HDV infection, indicating that Ntcp is the only restricting factor for HBV/HDV infection of macaque hepatocytes.

**Fig 6:**
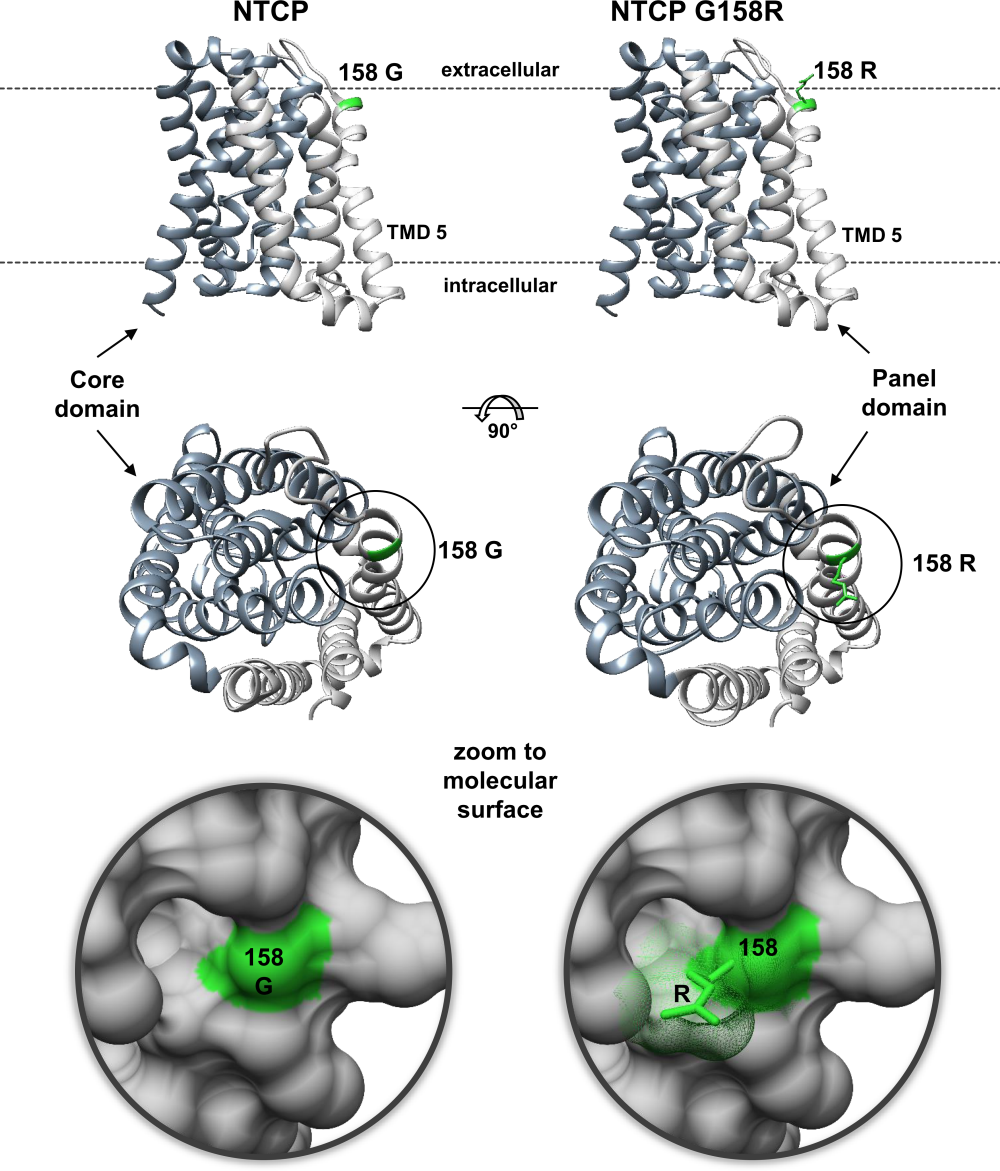
Localization of amino acid 158 at human NTCP. Homology model of human NTCP based on the crystal structure of ASBT_Yf_ [22]. The model represents amino acids 27–308 of NTCP. The panel domain is depicted in light and the core domain in dark grey. Localisation of the protein within the plasma membrane is indicated by dashed lines and was calculated based on transmembrane domain predictions performed with HMMTOP and MEMSAT3. Amino acid 158 is highlighted in green. Replacement of glycine 158 with the more bulky arginine abolished myr-preS1-peptide binding and *in vitro* infection, probably by sterically blocking the virus attachment site of NTCP.

The significant role of arginine 158 in providing HBV/HDV insensitivity gives reason to further speculation. As this arginine is conserved among all analysed OWM Ntcps it could have been occurred by mutation early in the branch of *Cercopithecidae*. One can speculate that this mutation was a chance event. On the other hand G158R mutation might have occurred in answer to a particular selection pressure, probably due to endemic hepadnavirus infection with pathological significance in a more ancient *Cercopithecidae* population. Apart from this evolutionary aspect our findings can be of relevance for the development of HBV/HDV entry inhibitors that specifically block the virus attachment site of NTCP without hampering the bile salt transport function. As it is known that the bile salt transport and the myr-preS1 receptor functions of NTCP are linked to each other [4], preS1-peptide-derived HBV/HDV entry inhibitors such as Myrcludex B are expected to interfere with the bile salt homeostasis due to their mode of action. Indeed, recent application of Myrcludex B to healthy human volunteers dramatically increased the serum levels of conjugated bile salts by two orders of magnitude [23]. Therefore, specific blocking of the virus attachment site with a small(er) molecule might be beneficial. Following this direction, our data clearly show that a relatively small steric mass at position 158, irrespective of formed by the side chains of arginine, aspartate, asparagine, or valine, is sufficient to completely block myr-preS1-peptide binding to NTCP and HBV infection (Figs 3–5). If this space can be occupied by a small molecule targeting amino acid 158, it should be possible to block viral binding via its myr-preS1-peptide domain without interfering with the bile salt transport. Based on a 3D homology model of human NTCP, amino acid 158 is located at a extracellular accessible site of NTCP, directly preceding membrane insertion of transmembrane domain 5 (Fig 6). It was suggested that bile salt transport is facilitated by movement of a flexible panel domain (TMDs 1, 5, 6) against a more rigid core domain (TMDs 2–4 and 7–9) [5]. Interestingly, amino acid 158 is located near the proposed entry site for bile salts, what would structurally explain, why bile salts block myr-preS1-peptide binding to Ntcp [4]. Furthermore, it can be hypothesised that myr-preS1-binding clamps NTCP in the depicted inward open conformation (Fig 6) by attachment to amino acid 158 and additional less-specific binding sites at the core domain. So, the myr-preS1-peptide would block the flexible movements necessary for the bile salt transport. In contrast, a small specific inhibitor of the viral attachment site at amino acid 158 of Ntcp would allow such flexible movements and so would not interfere with bile salt transport. This hypothesis is supported by recent findings with the cyclic peptide cyclosporine A that blocks myr-preS1-peptide binding and bile salt transport function of NTCP [24]. But a KG157-158GR mutant of NTCP became preS1-binding deficient, but still supported bile salt transport, similar to the G158R mutants of the present study. This transport of the mutant was insensitive to cyclosporine A, indicating that amino acids 157–158 represent the target site of cyclosporine A. Other cyclosporine derivatives (SCY450 and SCY995) were even unreactive at the bile salt transport of wild type NTCP, although they blocked myr-preS1-peptide binding and HBV infection [25]. These data are encouraging that further small molecules can be identified that fit the proposed profile.

Interestingly, the myr-preS1-peptide binding capability of the R158G OWM Ntcp mutants reached values similar to the wild type NTCP/Ntcps of *Hominidae* (Fig 4). However, the ability of these R158G mutants to support HBV/HDV infection was 10 times lower (Fig 5). This most likely is determined by sequence variations in further domains of Ntcp. Amino acids 84–87 are candidates in this regard as they were previously identified to be critical for internalisation of the virus/receptor complex and subsequent HBV/HDV infection [3,14]. Compared to the sequence of human NTCP (being “RLKN”), OWM Ntcps all bear a “QLNN” sequence at the corresponding position (Fig 2). In contrast, amino acid position 263, which was recently described to be crucial for viral entry [26] is highly conserved among all analysed NTPC/Ntcps (except for S.sci) and so seems not to be of relevance here.

In the group of cloned NWM Ntcps, three different susceptibility pattern were detected: the Ntcps of S.sci and C.jac, both with glycine at position 158, showed clear binding of the HBV/WMHBV myr-preS1 peptides. Only S.sci Ntcp-transfected HepG2 cells showed faint susceptibility for HBV infection, while the Ntcp from S.oed was completely insensitive for myr-preS1-peptide binding and HBV infection. This heterogeneous susceptibility pattern needs further investigation that should include even more Ntcps from different *Platyrrhini* families and species. Phylogenetically, there are five *Platyrrhini* families: *Callitrichidae*, *Cebidae*, *Aotidae*, *Pitheciidae*, and *Atelidae*. Hepadnaviruses have been isolated so far from woolley monkey [8] (family *Atelidae*) and very recently from capuchin monkey [9] (family *Cebidae*), while Ntcps have been cloned in the present study from the families *Callitrichidae* (C.jac and S.oed) as well as *Cebidae* (S.sci). Despite of this limited knowledge, the present study showed for the first time *in vitro* infection of HBV via Ntcp from the NWM *Saimiri sciureus*, demonstrating the potential for transmission of HBV to certain, but probably not all NWM.

## Conclusions

Amino acid position 158 of NTCP/Ntcp is sufficient to discriminate between the HBV/HDV susceptible group of humans and great apes (158G) and the non-susceptible group of OWM (158R). Thus, at the level of viral binding and entry via Ntcp, the observed species barriers do not appear as large as it was generally assumed. In the group of NWMs, Ntcps exhibit higher sequence variability and more heterogenous HBV/WMHBV susceptibility pattern that needs further systematic investigation and inclusion of even more Ntcps from *Ceboidea* species to better understand the virus-host determinants in this branch of nonhuman primates. Cloning of Ntcps from NWM in the present study facilitated for the first time *in vitro* infection of HBV via Ntcp from a NWM, the common squirrel monkey (*Saimiri sciureus*).

## Supporting information

S1 Fig Full sequence alignment of the cloned monkey Ntcps. The deduced amino acid sequences of all cloned monkey Ntcps from apes (black, 1–3), Old World monkeys (orange, 4–9), and New World monkeys (red, 10–12) were aligned with the EBI Clustal Omega algorithm (https://www.ebi.ac.uk), and the alignment was visualised by BOXshade (https://embnet.vital-it.ch). Amino acid identity is displayed with black shading, and amino acid similarities are highlighted in grey. Gaps (-) are introduced to optimise the alignment. All cloned sequences were deposited into the DDBJ/ENA/GenBank database with the Accession numbers listed in Table 1. Full species names are given in Table 3. NTCP/Ntcps from non-primate species are included for comparison. Regions critical for HBV binding (light green) and infection (dark green) are indicated. Amino acid position 158 is additionally marked by “X”. Highly conserved N-glycosylation sites in the N-terminus of all NTCP/Ntcps are indicated by “Y”. Localisation of the transmembrane domains, derived from the homology model of human NTCP (see Figs 2 and 6), are marked by TMD-I to TMD-IX. Whereas most monkey Ntcps have a highly conserved C-terminal end, the C-termini from C.jac and S.oed are elongated by frameshift at amino acid position 345. These elongated C-termini have no sequence similarity with any of the non-primate Ntcp sequences at all.
(DOCX)

S2 Fig *Papio hamadryas* (8) and *Macaca silenus* (6) Ntcps: Transport, peptide binding and infection data. (**A**) HEK293 cells were transiently transfected with P.ham and M.sil Ntcp wild type or R158G mutant constructs. Transport activity was verified with NBD-TC (green fluorescence, nuclei blue fluorescence) and [^3^H]TC. Absence of myr-preS1 served as positive control (scaled to 100%, open bars). HBV or WMHBV myr-preS1-peptides served as inhibitors at increasing concentrations. Negative control: uptake in sodium-free buffer (black bars). Data represent means ± SD of n = 3 determinations. ^#^Significant transport inhibition compared to positive control, *p*<0.0001. *Significantly different from the corresponding value of the wild type Ntcp, *p*<0.001 (two-way ANOVA). (**B**) Transfected HEK293 cells were incubated with the fluorescently labelled myr-preS1-HBV-Al633 (blue columns) or myr-preS1-WMHBV-Al633 (red columns) peptides (10 nM, 20 min, 37°C). After washing, cells were analysed for Al633 fluorescence. Data represent means ± SD of three combined independent experiments each with triplicate determinations. Representative fluorescence scans are shown for myr-preS1-HBV-Al633 (left half) and myr-preS1-WMHBV-Al633 (right half). SOAT-expressing HEK293 cells served as negative control, as they are unable to bind the myr-preS1-peptide [4]. ^*^Statistically different from the wild type clone with *p*<0.001 (two-way ANOVA). ^#^No significant difference to negative control with *p*<0.001. Rfu, relative fluorescence units. (**C**) HepG2 cells were transiently transfected with the P.ham and M.sil Ntcp wild type or R158G mutant constructs in 96 well plates and inoculated with 10,000 genome equivalents of pseudotyped HDV particles (HDVpsHBV/HDVpsWMHBV) or 2,000 HBV genome equivalents per cell. Cells were cultured for 10 days post infection and then immunostained against HDAg or HBcAg. Infected cells per well were manually counted by fluorescence microscopy. Cell counts are depicted as means ± SD of one representative experiment performed in triplicate.
(TIF)

S3 Fig Time course of anti-HBcAg immunostaining signals after HBV infection in NTCP-transfected HepG2 cells. HepG2 cells were transiently transfected with human NTCP in 96 well plates and inoculated with 2,000 HBV genome equivalents per cell. Cells were cultured and immunostaining against the HBcAg (red fluorescence) was performed at days 4, 7, and 10 post infection. Nuclei were counterstained with DAPI (blue fluorescence). Bars represent 20 µm. While no specific anti-HBcAg immunofluorescence could be detected at day 4, there were significant signals at day 7, which further increased in intensity at day 10.
(TIF)

S4 Fig Localisation of amino acid 158 at *Macaca mulatta* Ntcp. Homology model of M.mul Ntcp based on the crystal structure of ASBT_Yf_ (PDB 4n7w [22]). The model covers amino acids 28 to 308 of the M.mul Ntcp protein with an outward orientation of the N-terminus and an intracellular localisation of the C-terminus. The panel domain is depicted in light and the core domain in dark grey. The native amino acid arginine 158 is highlighted in green in the M.mul Ntcp. Mutation to glycine with its small hydrogen side chain seems to reopen the sterically blocked HBV binding domain and so M.mul Ntcp again becomes sensitive for myr-preS1-peptide binding and susceptible for HBV/HDV infection. Interestingly, amino acid 158 is located near the proposed entry site for bile salts, which pass through the NTCP most likely at the interface between the core and the panel domains. This would structurally explain, why bile salts block myr-preS1-peptide binding to Ntcp and *vice versa* (see [4]). Based on this model Old World monkeys could have become unsusceptible for HBV/HDV infection by a relatively small structural modification at the viral attachment site of Ntcp.
(TIF)
