# Supplementary material for: Characterisation of the hepatitis B virus cross-species transmission pattern via Na+/taurocholate co-transporting polypeptides from 11 New World and Old World primate species
Source: PLoS One. 2018 Jun 18;13(6):e0199200. doi: 10.1371/journal.pone.0199200 (PMC6005513; doi:10.1371/journal.pone.0199200)
Supplement: S1 Fig — The deduced amino acid sequences of all cloned monkey Ntcps from apes (black, 1–3), Old World monkeys (orange, 4–9), and New World monkeys (red, 10–12) were aligned with the EBI Clustal Omega algorithm (https://www.ebi.ac.uk), and the alignment was visualised by BOXshade (https://embnet.vital-it.ch). Amino acid identity is displayed with black shading, and amino acid similarities are highlighted in grey. Gaps (-) are introduced to optimise the alignment. All cloned sequences were deposited into the DDBJ/ENA/GenBank database with the Accession numbers listed in Table 1. Full species names are given in Table 3. NTCP/Ntcps from non-primate species are included for comparison. Regions critical for HBV binding (light green) and infection (dark green) are indicated. Amino acid position 158 is additionally marked by “X”. Highly conserved N-glycosylation sites in the N-terminus of all NTCP/Ntcps are indicated by “Y”. Localisation of the transmembrane domains, derived from the homology model of human NTCP (see Figs 2 and 6), are marked by TMD-I to TMD-IX. Whereas most monkey Ntcps have a highly conserved C-terminal end, the C-termini from C.jac and S.oed are elongated by frameshift at amino acid position 345. These elongated C-termini have no sequence similarity with any of the non-primate Ntcp sequences at all. (DOCX) [file pone.0199200.s001.docx]

**S1 Figure**

**N-terminus**

**Y Y -------TMD-I------- ---**

**H.sap (1) 1 MEAHNASAPFNFTLPPNFGKRPTDLALSVILVFMLFFIMLSLGCTMEFSKIKAHLWKPKG |**

**P.tro (2) 1 MEAHNVSAPFNFTLPPNFGKRPTDLALSVILVFMLFFIMLSLGCTMEFSKIKAHLWKPKG | apes**

**P.abe (3) 1 MEAHNASAPFNFTLPPNFGKRPTDLALSVILVFMLFFIMLSLGCTMEFSKIKAHLWKPKG |**

**C.aet (4) 1 MEAHNASAPFNFTLPPNFGKRPTDLALSIILVFMLFFVMLSLGCTMEFSKIKAHLWKPKG |**

**M.mul (5) 1 MEAHNASAPFNFTLPPNFGKRPTDLALSIILVFMLFFVMLSLGCTMEFSKIKAHLWKPKG | Old
M.sil (6) 1 MEAHNASAPFNFTLPPNFGKRPTDLALSIILVFMLFFVMLSLGCTMEFSKIKAHLWKPKG | World
M.fas (7) 1 MEAHNASAPFNFTLPPNFGKRPTDLALSIILVFMLFFVMLSLGCTMEFSKIKAHLWKPKG | monkeys
P.ham (8) 1 MEAHNASAPFNFTLPPNFGKRPTDLALSIILVFMLFFVMLSLGCTMEFSKIKAHLWKPKG |**

**S.spe (9) 1 MEAHNTSAPFNFTLPPNFGKRPTDLALSIILVFMLFFVMLSLGCTMEFSKIKAHLWKPKG |
S.sci (10) 1 MDAHNMSATFNFTLPPNFGKRPTDLALSIILVFMLFFIMLSLGCTMEFSKIKAHFWKPKG | New
C.jac (11) 1 MEAHNVSATFNFTLPPDFGKRPTDLALSIILVFMLFFIMLSLGCTMEFSKIKAHFWKPKG | World
S.oed (12) 1 MEAHNVSATFNFTLPPNFGKRPTDLALSIILVFMLFFIMLSLGCTMEFSKIKAHFWKPKG | monkeys
T.bel 1 MEAHNLSAPLNFTLPPNFGKRPTDQALSVILVVMLLIMMLSLGCTMEFSKIKAHFWKPKG**

**C.lup 1 MDAPNITAPLNFTLPPNFGKRPTDKALSIILVFLLLIIMLSLGCTMEFSKIKAHFWKPKG**

**R.nor 1 MEVHNVSAPFNFSLPPGFGHRATDKALSIILVLMLLLIMLSLGCTMEFSKIKAHLWKPKG
M.mus 1 MEAHNVSAPFNFSLPPGFGHRATDTALSVILVVMLLLIMLSLGCTMEFSKIKAHFWKPKG
S.scr 1 MEALNESAPINFTLPHNFGKRPTDLALSVILVFMLLIIMLSLGCTMEFGRIRAHFRKPKG**

**-------TMD-II----------...------------TMD-III-------- -----
H.sap (1) 61 LAIALVAQYGIMPLTAFVLGKVFRLKNIEALAILVCGCSPGGNLSNVFSLAMKGDMNLSI**

**P.tro (2) 61 LAIALVAQYGIMPLTAFVLGKVFRLKNIEALAILVCGCSPGGNLSNVFSLAMKGDMNLSI**

**P.abe (3) 61 LAIALVAQYGIMPLTAFVLGKVFRLKNIEALAILVCGCSPGGNLSNVFSLAMKGDMNLSI**

**C.aet (4) 61 LAIALVAQYGIMPLTAFVLGKVFQLNNIEALAILVCGCSPGGNLSNVFSLAMKGDMNLSI**

**M.mul (5) 61 LAIALVAQYGIMPLTAFVLGKVFQLNNIEALAILVCGCSPGGNLSNVFSLAMKGDMNLSI
M.sil (6) 61 LAIALVAQYGIMPLTAFVLGKVFQLNNIEALAILVCGCSPGGNLSNVFSLAMKGDMNLSI
M.fas (7) 61 LAIALVAQYGIMPLTAFVLGKVFQLNNIEALAILVCGCSPGGNLSNVFSLAMKGDMNLSI
P.ham (8) 61 LAIALVAQYGIMPLTAFVLGKVFQLNNIEALAILVCGCSPGGNLSNVFSLAMKGDMNLSI**

**S.spe (9) 61 LAIALVAQYGIMPLTAFVLGKVFQLNNIEALAILVCGCSPGGNLSNVFSLAMKGDMNLSI
S.sci (10) 61 LAIALVAQYGIMPLTAFVLGKVFQLNKIEALAILVCGCSPGGNLSNVFSLAMKGDMNLSI
C.jac (11) 61 LAIALVAQYGIMPLTAFVLGKVFRLNKIEALAILVCGCSPGGTLSNVFSLAMKGDMNLSI
S.oed (12) 61 LAIALVAQYGIMPFTAFMLGKVFLLNKTEALAILVCGCSPGGTLSNVFSLAMKGDMNLSI
T.bel 61 LAIALLAQYGIMPLTAFALGKVFPLNNIEALAILVCGCSPGGNLSNVFSLAMKGDMNLSI
C.lup 61 LVIALIAQYGIMPLTAFTLGKVFRLNNIEALAILVCGCSPGGTLSNVFSLAMKGDMNLSI
R.nor 61 VIVALVAQFGIMPLAAFLLGKIFHLSNIEALAILICGCSPGGNLSNLFTLAMKGDMNLSI
M.mus 61 VIIAIVAQYGIMPLSAFLLGKVFHLTSIEALAILICGCSPGGNLSNLFTLAMKGDMNLSI
S.scr 61 LAIALVAQYGIMPLTAFALGKLFRLNNVEALAILICGCSPGGNLSNIFALAMKGDMNLSI**

**158
 -----TMD-IV---------- --X--------TMD-V--------
H.sap (1) 121 VMTTCSTFCALGMMPLLLYIYSRGIYDGDLKDKVPYKGIVISLVLVLIPCTIGIVLKSKR**

**P.tro (2) 121 VMTTCSTFCALGMMPLLLYIYSRGIYDGDLKDKVPYKGIVISLVLVLIPCTIGIVLKSKR**

**P.abe (3) 121 VMTTCSTFCALGMMPLLLYIYSRGIYDGDLKDKVPYRGIVISLVLVLIPCTIGIVLKSKR**

**C.aet (4) 121 VMTTCSTFCALGMMPLLLYLYTRGIYDGDLKDKVPYGRIILSLVPVLIPCTIGIVLKSKR**

**M.mul (5) 121 VMTTCSTFCALGMMPLLLYLYTRGIYDGDLKDKVPYGRIILSLVPVLIPCTIGIVLKSKR
M.sil (6) 121 VMTTCSTFCALGMMPLLLYLYTRGIYDGDLKDKVPYGRIILSLVPVLIPCTIGIVLKSKR
M.fas (7) 121 VMTTCSTFCALGMMPLLLYLYTRGIYDGDLKDKVPYGRIILSLVPVLIPCTIGIVLKSKR
P.ham (8) 121 VMTTCSTFCALGMMPLLLYLYTRGIYDGDLKDKVPYGRIILSLVPVLIPCTIGIILKSKR
S.spe (9) 121 VMTTCSTFCALGMMPLLLYLYSRGIYDGDLKDKVPYGRIILSLVPVLIPCTIGIVLKSKR
S.sci (10) 121 VMTTCSTFCALGMMPLLLYIYSRGIYDGDLKDKVPYGGIMISLILVLIPCTIGIVLKSKR
C.jac (11) 121 VMTTCSTFCALGMMPLLLYIYSRGIYEGDLKDKVPYKGIMRSLILVLIPCTIGIILKSKR
S.oed (12) 121 VMTTCSTFCALGMMPLLLYIYSTGIYEGDLKDKVPYKSIMISLILVLIPCTIGIVLKSKR
T.bel 121 VMTTCSTFFALGMMPLLLYIYSKGIYDGDLKDKVPYVGIVISLILVLIPCTIGIFLKSKR
C.lup 121 VMTTCSTFFALGMMPLLLYIYSNGIYDGDLKDKVPYKGIVSSLVLVLIPCTIGIFLKAKR
R.nor 121 VMTTCSSFSALGMMPLLLYVYSKGIYDGDLKDKVPYKGIMISLVIVLIPCTIGIVLKSKR
M.mus 121 VMTTCSSFTALGMMPLLLYIYSKGIYDGDLKDKVPYKGIMLSLVMVLIPCAIGIFLKSKR
S.scr 121 MMTTCSTFLALGMMPLLLYLYSRGIYDGTLKDKVPYGSIVISLILILIPCTIGIILNTKR**

**-------------TMD-VI----------- ---------TMD-VII-------------
H.sap (1) 181 PQYMRYVIKGGMIIILLCSVAVTVLSAINVGKSIMFAMTPLLIATSSLMPFIGFLLGYVL**

**P.tro (2) 181 PQYMRYVIKGGMIIILLCSVAVTVLSAINVGKSIMFAMTPLLIATSSLMPFIGFLLGYVL**

**P.abe (3) 181 PQYMRYVIKGGMIIILLCSVAVTVLSAINVGKSIMFAMTPLLIATSSLMPFIGFLLGYVL**

**C.aet (4) 181 PQYMRYVIKGGMIIILLCSVAVTVLSAINVGKSIMFAMTPLLIATSSLMPFIGFLLGYVL
M.mul (5) 181 PQYMRYVIKGGMIIILLCSVAVTVLSAINVGKSIMFAMTPLLIATSSLMPFIGFLLGYVL
M.sil (6) 181 PQYMRYVIKGGMIIILLCSVAVTVLSAINVGKSIMFAMTPLLIATSSLMPFIGFLLGYVL
M.fas (7) 181 PQYMRYVIKGGMIIILLCSVAVTVLSAINVGKSIMFAMTPLLIATSSLMPFIGFLLGYVL
P.ham (8) 181 PQYMRYVIKGGMIIILLCSVAVTVLSAINVGKSIMFAMTPLLIATSSLMPFIGFLLGYVL**

**S.spe (9) 181 PQYMRYVIKGGTIIILLCSVAVTVLSAINVGKSIMFAMTPVLIATSSLMPFIGFLLGYVL**

**S.sci (10) 181 PQYVRYVVKGGMIIILLCSVTVIVLSAINVGKSILFAMTPLLVTTSSLMPFIGFLLGYVL
C.jac (11) 181 PQYVRYVVKGGMIIILLCSVAVIVLSAINVGKSILLAMTPLLVTTSSLMPFIGFLLGYVL
S.oed (12) 181 PQYVRYVVKGGMIIVLLCSVAVIVLSAINVGKSILFAMTPLLVTTSSLMPFIGFLLGYVL
T.bel 181 PQYVPYVTKAGMIIILLLSVAITVLSVINVGKSIMFVMTPHLLATSSLMPFIGFLLGYIL
C.lup 181 PQYVRYIKKGGMIIMLLLSVAITALSVINVGKSIRFVMTPHLLATSSLMPFIGFLLGYIL
R.nor 181 PHYVPYILKGGMIITFLLSVAVTALSVINVGNSIMFVMTPHLLATSSLMPFSGFLMGYIL
M.mus 181 PHYVPYVLKAGMIITFSLSVAVTVLSVINVGNSIMFVMTPHLLATSSLMPFTGFLMGYIL
S.scr 181 PQYVRYVIKGGTILLILCAIAVTVLSVLNVGKSILFVMTPHLVATSSLMPFTGFLLGYLL**

**--- ---------TMD-VIII--------- -------TMD-IX---
H.sap (1) 241 SALFCLNGRCRRTVSMETGCQNVQLCSTILNVAFPPEVIGPLFFFPLLYMIFQLGEGLLL**

**P.tro (2) 241 SALFCLNGRCRRTVSMETGCQNVQLCSTILNVAFPPEVIGPLFFFPLLYMIFQLGEGLLL**

**P.abe (3) 241 SALFCLNGRCRRTVSMETGCQNVQLCSTILNVAFPPEVIGPLFFFPLLYMIFQLGEGLLL**

**C.aet (4) 241 SALFCLNGRCRRTVSMETGCQNVQLCSTILNVAFPPEVIGPLFFFPLLYMIFQLGEGLLL**

**M.mul (5) 241 SALFCLNGRCRRTVSMETGCQNVQLCSTILNVAFPPEVIGPLFFFPLLYMIFQLGEGLLL
M.sil (6) 241 SALFCLNGRCRRTVSMETGCQNVQLCSTILNVAFPPEVIGPLFFFPLLYMIFQLGEGLLL
M.fas (7) 241 SALFCLNGRCRRTVSMETGCQNVQLCSTILNVAFPPEVIGPLFFFPLLYMIFQLGEGLLL
P.ham (8) 241 SALFCLNGRCRRTVSMETGCQNVQLCSTILNVAFPPEVIGPLFFFPLLYMIFQLGEGLLL**

**S.spe (9) 241 SALFCLNARCRRTVSMETGCQNVQLCSTILNVAFPPEVIGPLFFFPLLYMIFQLGEGLFL
S.sci (10) 241 SALFCLNGRCRRTVSMETGCQNIQLCSTILNVAFPPEVIGPLFFFPLLYMIFQLGEGLLL
C.jac (11) 241 CALFCLNGRCRRTVSMETGCQNVQLCSTILNVAFPPEVIGPLFFFPLLYMIFQLGEGLLL
S.oed (12) 241 SALFCLNGRCRRTVSMETGCQNVQLCSTILNVAFPPEVIGPLFFFPLLYMIFQLGEGLLF
T.bel 241 STLFRLNAQCSRTVSMETGCQNVQLCSTILNVTFRPEVIGPLFFFPLLYMIFQLGEGLLL**

**C.lup 241 SALFRLDGRCSRTVSMETGCQNVQLCSTILNVTFPPEVIGPLFFFPLLYMIFQLGEGVFL**

**R.nor 241 SALFQLNPSCRRTISMETGFQNIQLCSTILNVTFPPEVIGPLFFFPLLYMIFQLAEGLLI
M.mus 241 SALFRLNPSCRRTISMETGFQNVQLCSTILNVTFPPEVIGPLFFFPLLYMIFQLAEGLLF
S.scr 241 SALFRLNARCSRTVCMETGCQNVQLCSTILNVTFPPEVIGPLFFFPLLYMLFQLGEGLLF**

**C-terminus**

**------
H.sap (1) 301 IAIFWCYEKFKTPKDKTKMIYTAATTEETIPGALGNGTYKGEDCSPCTA-----------**

**P.tro (2) 301 IAMFWCYEKFKTPKDKTKMTYTAATTEETIPGALGNGTYKGEDCSPCTA-----------**

**P.abe (3) 301 IAMFWCYEKFKTPKGKTKMIYTAATTEETIPGALGNGTYKGEDCSPCTA-----------**

**C.aet (4) 301 IAMFRCYEKFKTPKDKTKMIYTAATTEETIPGALGNGTYKGEDCSPCTA-----------
M.mul (5) 301 IAMFRCYEKFKTPKDKTKMIYTAATTEETIPGALGNGTYKGEDCSPCTA-----------
M.sil (6) 301 IAMFRCYEKFKTPKDKTKMIYTAATTEETIPGALGNGTYKGEDCSPCTA-----------
M.fas (7) 301 IAMFRCYEKFKTPKDKTKMIYTAATTEETIPAALGNGTYKGEDCSPCTA-----------
P.ham (8) 301 IVMFRCYEKFKTPKDKTKMIYTAATTEETIPGALGNGTYKGEDCSPCTA-----------**

**S.spe (9) 301 IAIFRCYEKFKTPKDKTKMIYTAATTEETIPGALGNGTYKGEDCSPCTA-----------
S.sci (10) 301 IAMFRCYEKFKTPKDKTKIIYTAATSEETTPGAVGNGTYKGKECSPCKA-----------
C.jac (11) 301 IAMFRCYEKFKTPKDKMKIIYTAATTEETTPGAVGNGIYKGKECSLAEPSPSHGGLDSGP
S.oed (12) 301 IAMFRCYEKFKTPKDKTKIIYTAATTEETTPGAVKNGIYKGKECSLAEPSPSHGSLDSGP
T.bel 301 IAIYRCYEKIKTSKDKTKVIYTAAKTEETIPGTLGNSTHKCEEYSPYTVENSTHKCEEYS
C.lup 301 ISIFRCYEKIKPSKDKTKMIYTAAATEEITPGALGNGTHKGEECSPCTAAPSPSGLDSGE**

**R.nor 301 IIIFRCYEKIKPPKDQTKITYKAAATEDATPAALEKGTHNGN-IPPLQPGPSPNGLNSGQ
M.mus 301 IIIFRCYLKIKPQKDQTKITYKAAATEDATPAALEKGTHNGN-NPPTQPGLSPNGLNSGQ
S.scr 301 IAIFRCYEKTKLSKDKMKTISAADSTEETIPTALGNGTHKGEECPPTQPSVV--------

H.sap (1) --------------------------------**

**P.tro (2) --------------------------------**

**P.abe (3) --------------------------------**

**C.aet (4) --------------------------------**

**M.mul (5) --------------------------------
M.sil (6) --------------------------------
M.fas (7) --------------------------------
P.ham (8) --------------------------------**

**S.spe (9) --------------------------------**

**S.sci (10) --------------------------------
C.jac (11) 361 KANSESQSGKLERAAKTPVLPESLQHF-----
S.oed (12) 361 KANSKSQSGKL---------------------**

**T.bel 361 PSTVGNGTYKGEECSPGTA-------------**

**C.lup 361 KAIQCDQLEKAKDKRNTKEESFSSIGSSNYQN**

**R.nor 360 MAN-----------------------------
M.mus 360 MAN-----------------------------
S.scr --------------------------------**
