# Supplementary figures and images for: Characterisation of the hepatitis B virus cross-species transmission pattern via Na+/taurocholate co-transporting polypeptides from 11 New World and Old World primate species
Source: PLoS One. 2018 Jun 18;13(6):e0199200. doi: 10.1371/journal.pone.0199200 (PMC6005513; doi:10.1371/journal.pone.0199200)

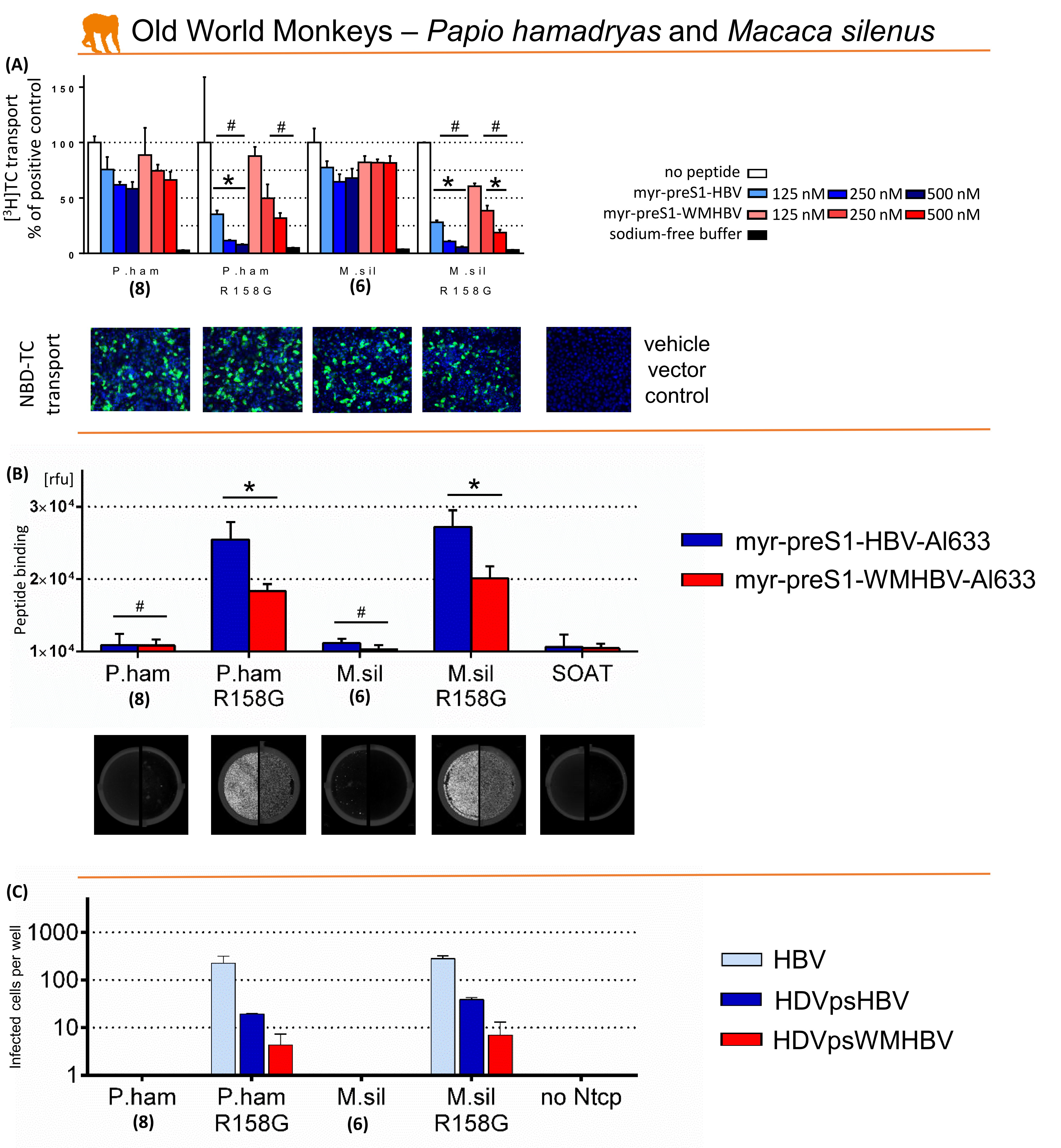

Supplement: S2 Fig — (A) HEK293 cells were transiently transfected with P.ham and M.sil Ntcp wild type or R158G mutant constructs. Transport activity was verified with NBD-TC (green fluorescence, nuclei blue fluorescence) and [3H]TC. Absence of myr-preS1 served as positive control (scaled to 100%, open bars). HBV or WMHBV myr-preS1-peptides served as inhibitors at increasing concentrations. Negative control: uptake in sodium-free buffer (black bars). Data represent means ± SD of n = 3 determinations. #Significant transport inhibition compared to positive control, p<0.0001. *Significantly different from the corresponding value of the wild type Ntcp, p<0.001 (two-way ANOVA). (B) Transfected HEK293 cells were incubated with the fluorescently labelled myr-preS1-HBV-Al633 (blue columns) or myr-preS1-WMHBV-Al633 (red columns) peptides (10 nM, 20 min, 37°C). After washing, cells were analysed for Al633 fluorescence. Data represent means ± SD of three combined independent experiments each with triplicate determinations. Representative fluorescence scans are shown for myr-preS1-HBV-Al633 (left half) and myr-preS1-WMHBV-Al633 (right half). SOAT-expressing HEK293 cells served as negative control, as they are unable to bind the myr-preS1-peptide [4]. *Statistically different from the wild type clone with p<0.001 (two-way ANOVA). #No significant difference to negative control with p<0.001. Rfu, relative fluorescence units. (C) HepG2 cells were transiently transfected with the P.ham and M.sil Ntcp wild type or R158G mutant constructs in 96 well plates and inoculated with 10,000 genome equivalents of pseudotyped HDV particles (HDVpsHBV/HDVpsWMHBV) or 2,000 HBV genome equivalents per cell. Cells were cultured for 10 days post infection and then immunostained against HDAg or HBcAg. Infected cells per well were manually counted by fluorescence microscopy. Cell counts are depicted as means ± SD of one representative experiment performed in triplicate. (TIF) [file pone.0199200.s002.tif]

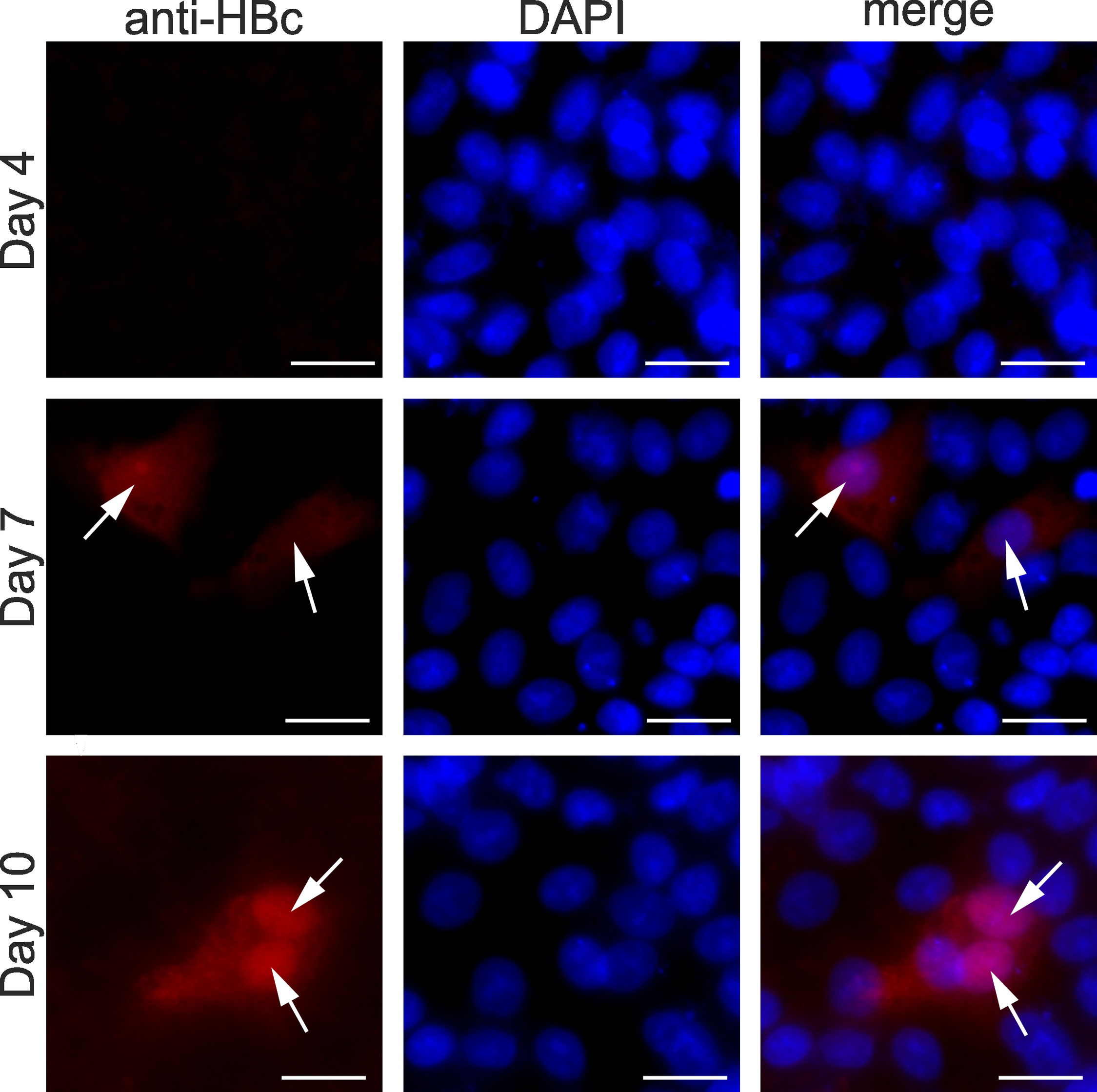

Supplement: S3 Fig — HepG2 cells were transiently transfected with human NTCP in 96 well plates and inoculated with 2,000 HBV genome equivalents per cell. Cells were cultured and immunostaining against the HBcAg (red fluorescence) was performed at days 4, 7, and 10 post infection. Nuclei were counterstained with DAPI (blue fluorescence). Bars represent 20 µm. While no specific anti-HBcAg immunofluorescence could be detected at day 4, there were significant signals at day 7, which further increased in intensity at day 10. (TIF) [file pone.0199200.s003.tif]

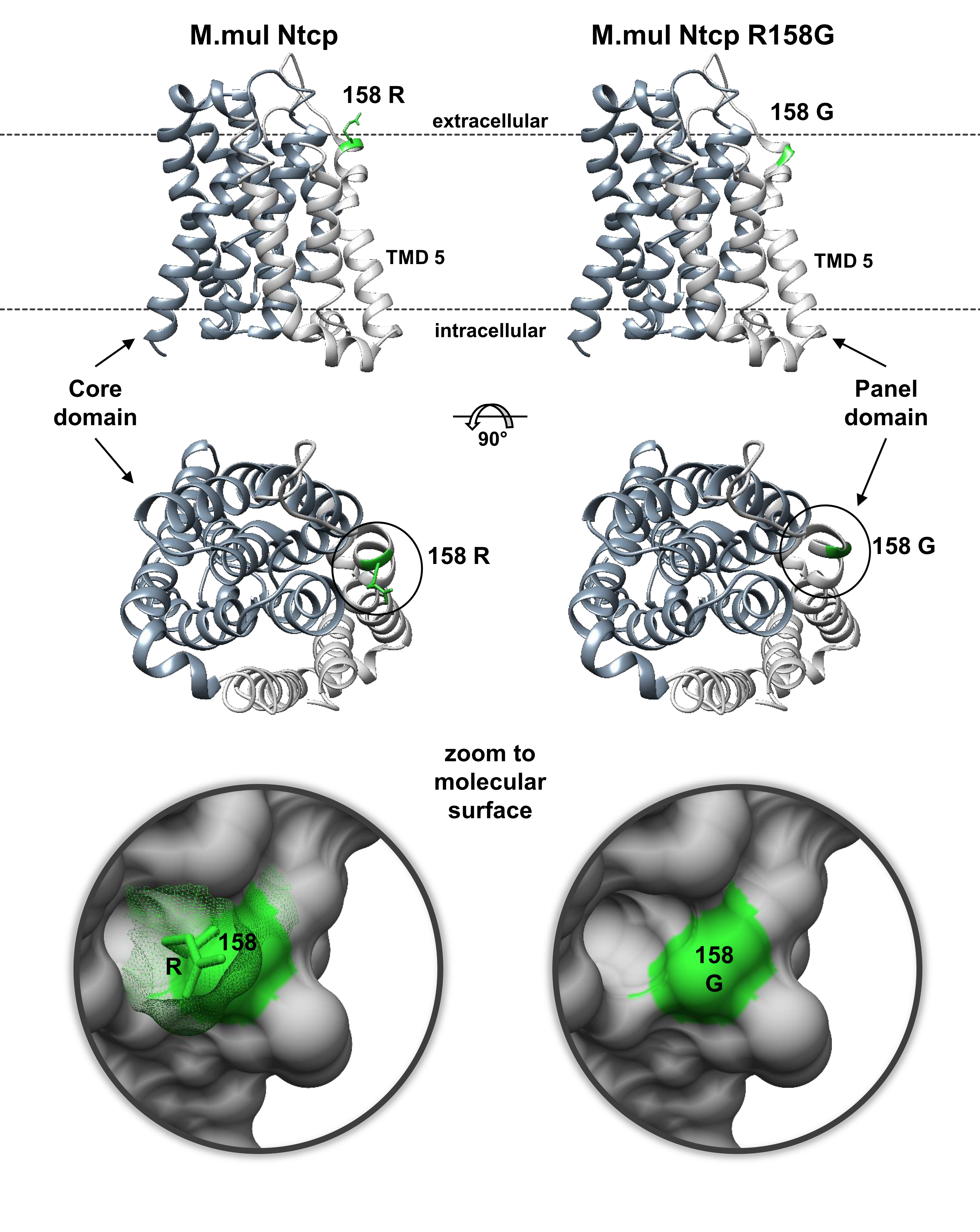

Supplement: S4 Fig — Homology model of M.mul Ntcp based on the crystal structure of ASBTYf (PDB 4n7w [22]). The model covers amino acids 28 to 308 of the M.mul Ntcp protein with an outward orientation of the N-terminus and an intracellular localisation of the C-terminus. The panel domain is depicted in light and the core domain in dark grey. The native amino acid arginine 158 is highlighted in green in the M.mul Ntcp. Mutation to glycine with its small hydrogen side chain seems to reopen the sterically blocked HBV binding domain and so M.mul Ntcp again becomes sensitive for myr-preS1-peptide binding and susceptible for HBV/HDV infection. Interestingly, amino acid 158 is located near the proposed entry site for bile salts, which pass through the NTCP most likely at the interface between the core and the panel domains. This would structurally explain, why bile salts block myr-preS1-peptide binding to Ntcp and vice versa (see [4]). Based on this model Old World monkeys could have become unsusceptible for HBV/HDV infection by a relatively small structural modification at the viral attachment site of Ntcp. (TIF) [file pone.0199200.s004.tif]
